# Molecular analysis of Aldh1a2 conditional inactivation in developing limbs reveals mechanisms of retinoic-acid mediated interdigital apoptosis

**DOI:** 10.3389/fcell.2026.1913173

**Published:** 2026-09-21

**Authors:** Rodrigue Peraldi, Yacine Kherdjemil, Gabriel Villiard, Nayuta Yakushiji-Kaminatsui, Marie Kmita

**Affiliations:** 1 Genetics and Development Research Unit, Institut de Recherches Cliniques de Montréal, Montréal, QC, Canada; 2 Département de Médecine (Programme de Biologie Moléculaire), Université de Montréal, Montréal, QC, Canada; 3 Plateforme Modélisation des Maladies et édition Du Génome, Institut de Recherches Cliniques de Montréal, Montréal, QC, Canada; 4 Laboratory for Developmental Genetics, RIKEN Center for Integrative Medical Sciences (IMS), Yokohama, Japan

**Keywords:** ALDH1A2, digit patterning, interdigital apoptosis, limb development, retinoic acid

## Abstract

**Introduction:**

Retinoic acid (RA) plays a plethora of functions during embryonic development as well as post-natal life. In particular, RA function during limb development is associated with interdigital cell death. However, the molecular mechanism(s) through which RA signaling contributes to interdigital cell death remains poorly understood.

**Methods:**

To gain insights into the mechanism(s) involved, we used a conditional approach to inactivate the RA catalyzing enzyme Aldh1a2 thereby abrogating RA signaling in the distal limb.

**Results and Discussion:**

Our data first show that *Aldh1a2* mutant limb buds are characterized by a lack of apoptosis in the interdigital region, which results in a very severe syndactyly phenotype at birth. Molecular analysis of *Aldh1a2* mutant distal limb buds reveals that RA regulates the expression of several key genes in the interdigital tissue related to apoptosis in the limb and to the extracellular matrix degradation. The decreased expression of these genes in *Aldh1a2* mutant limb buds correlates with a decreased activity of their putative regulatory elements bound by the retinoic acid receptors supporting that these genes are possibly direct RA targets. We also identified mild skeletal defects in *Aldh1a2* conditional mutant, as newborns have smaller digits. This latter phenotype is associated with the decreased expression of genes important for chondrocytes differentiation and proliferation, notably *Ihh*, *Foxc2* and *Igf1*. Finally, we show that the distal expression of *Aldh1a2* likely relies on the function of *Hoxa13* and *Hoxd13* establishing a potential cascade of molecular events responsible for the interdigit tissue regression.

## Introduction

Retinoic acid (RA) is a metabolite derived from vitamin A/retinol that plays crucial roles in many organs and tissues during embryogenesis and post-natal life. RA is synthesized in a two-step manner from its precursor. First, Retinol is converted to retinaldehyde by ADH and RDH enzymes, and second, oxidization of retinaldehyde by ALDH1A enzymes (*Aldh1a1*, *Aldh1a2* and *Aldh1a3*) give rise to RA (Duester, 2008). Once produced, RA can diffuse into cells and bind nuclear receptors (RARs) to regulate gene expression (Ghyselinck and Duester, 2019).

During limb development, forelimb bud initiation and early/proximal patterning relies on RA (Niederreither et al., 1999; Niederreither et al., 2002). *Aldh1a2* expressed from the lateral plate mesoderm synthesizes RA that diffuses in the limb bud to induce the expression of *Meis1/2* genes (Mercader et al., 2000), which are known to confer the proximal identity of the developing limb (Mercader et al., 1999). At later stages of limb development, when the different limb segments have been established, *Aldh1a2* is re-expressed in the autopod (Figure 1A) and is the only ALDH1A enzyme expressed in this tissue (Desanlis, Paul and Kmita, 2020). From E12.5 onwards, *Aldh1a2* expression is specifically restricted to the interdigital domain (Figure 1A). In turn, *Cyp26b1*, which encodes an enzyme that degrades RA, is expressed in the digit condensations, ensuring that RA is restricted to the interdigital area (Chimal-Monroy et al., 2011). Several studies have provided evidence that RA is required for the proper apoptosis of the interdigital tissue. Bead grafting experiments in chick limbs have shown that RA can induce apoptosis before the onset of physiological cell death (Rodriguez-Leon et al., 1999). Moreover, RA supplementation in mice to rescue the early lethality of *Aldh1a2* inactivation allowed the analysis of mutant hindlimbs at E13.5, which revealed significant reduction of apoptotic cells in the interdigital tissue (Zhao et al., 2010). Similarly, analysis of *Rdh10* mutant (also referred to as *trex*), which survive until E14.5, showed that there was no interdigital tissue regression in mutant hindlimbs (Cunningham et al., 2011). Of note, in both mutants, the forelimb phenotype could not be assessed as their development was stunt. While these studies provide evidence for RA being associated with interdigital tissue apoptosis, the underlying mechanism(s) remain elusive.

**FIGURE 1:**
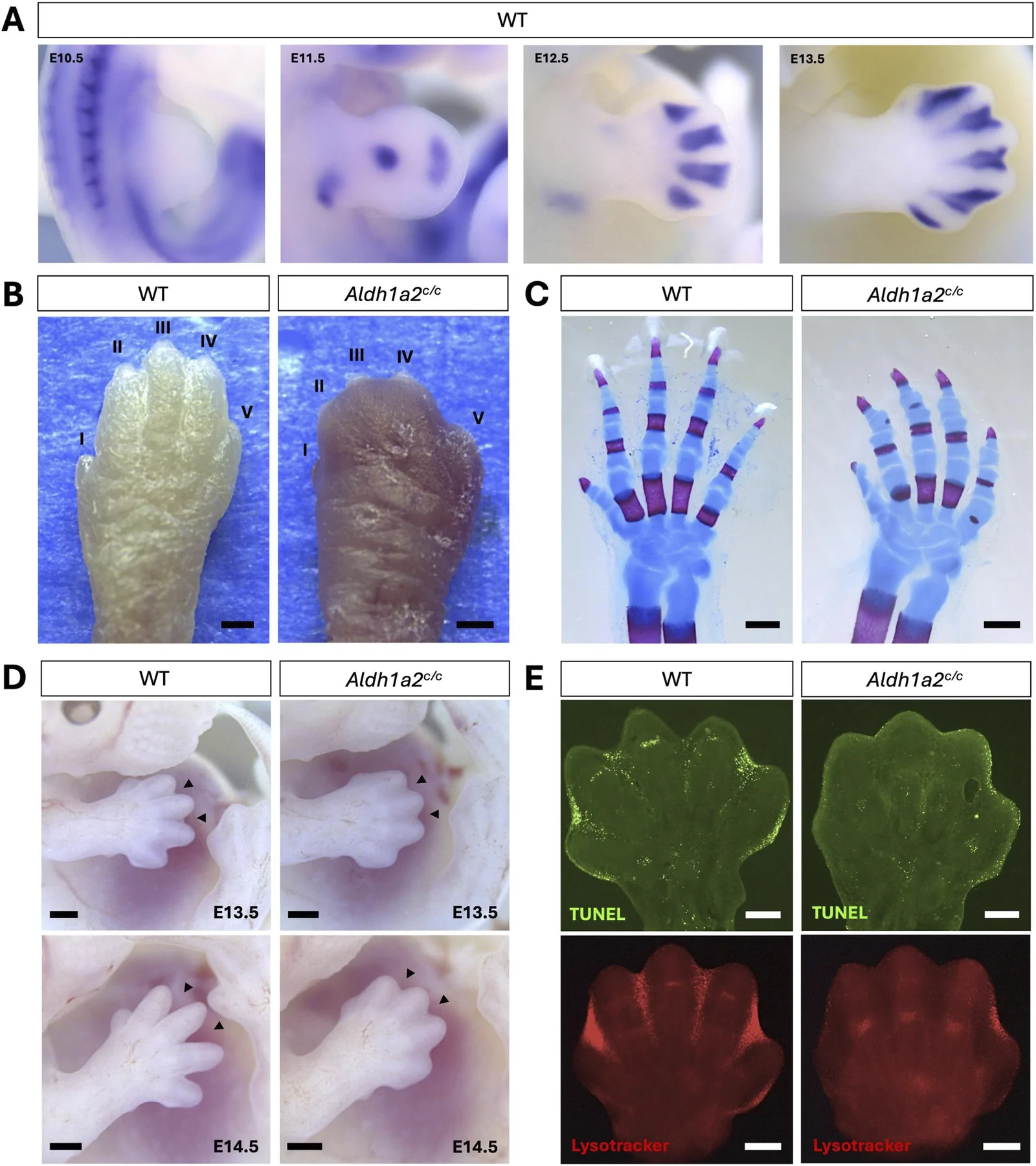
*Aldh1a2* ^*c/c*^ limbs are characterized by severe soft tissue syndactyly due to lack of apoptosis in the interdigit domains. **(A)** *Aldh1a2* expression pattern in WT embryonic forelimbs from E10.5 to E13.5 (n = 3). **(B)** Phenotype of WT and *Aldh1a2* ^*c/c*^ mutant autopods from newborn mice (n = 3). Scale bar is 500 µm. **(C)** Skeletons of WT and *Aldh1a2* ^*c/c*^ mutant autopods from newborn mice (n = 3). Scale bar is 500 µm. **(D)** Limb bud morphology of WT and *Aldh1a2* ^*c/c*^ mutant embryos at E13.5 (top) and E14.5 (bottom). Scale bar is 500 µm. **(E)** TUNEL (top) and Lysotracker (bottom) stainings on E13.5 forelimb of WT (n = 3) and *Aldh1a2* ^*c/c*^ mutant (n = 3) embryos. Scale bar is 250 µm.

To better understand the function of RA in the distal limb, we used a conditional approach to inactivate *Aldh1a2* specifically in the presumptive autopod using the *Hoxa13:Cre* deleter strain (Scotti et al., 2015). The resulting *Aldh1a2* conditional mutant displays a severe soft-tissue syndactyly phenotype, which is caused by the loss of interdigital apoptosis. By performing transcriptomic and genomic analyses, we reveal that RA regulates the expression of several genes important for proper interdigit tissue regression. In particular, RA regulates the expression of *Adamts9* and *Bmp7* and the Wnt inhibitor *Dkk1*, a gene known to be related to apoptosis (Grotewold and Rüther, 2002; Mukhopadhyay et al., 2001). *Aldh1a2* conditional inactivation also reveals impaired skeletal development, as digits of mutant newborns were smaller and display smaller primary ossification centers, which is likely due to the delay in chondrocyte differentiation toward hypertrophic chondrocytes.

## Materials and methods

### Mice

*Hoxa13* and *Hoxd13* line were previously reported (Fromental-Ramain et al., 1996).

To generate the conditional inactivation of *Aldh1a2* in the developing autopod, the *Aldh1a2* floxed line (Vermot et al., 2006) was crossed with the *Hoxa13:Cre* line (Scotti et al., 2015).

The *Eya2* knockout mouse line was generated using CRISPR-Cas9 system via the CRISPR-EZ procedure (Modzelewski et al., 2018). The targeting strategy was designed to delete exon 4, introducing an out-of-frame deletion predicted to result in a premature stop codon and loss of functional protein. Two sgRNAs targeting intronic sequences flanking exon 4 (sgRNA_intron3: TCC​GGA​GAG​TGT​AAT​GCC​TA and sgRNA_intron4: TGA​AGG​ACG​ATA​CAT​GTA​GC), were selected using IDT online CRISPR design tool and synthetized as chemically modified sgRNAs (IDT, Alt-R CRISPR-Cas9 sgRNA). Recombinant Cas9 nuclease (Alt-R™ S. p. HiFi Cas9 Nuclease V3, cat# 1081060) and the two sgRNAs were pre-assembled into ribonucleoprotein (RNP) complexes and electroporated into C57BL/6 J zygote embryos. Electroporated embryos were subsequently transferred to the oviducts of pseudopregnant CD-1 surrogate mothers. Founder animals were screened by PCR and Sanger sequencing to confirm the expected exon four deletion and resulting frameshift. Confirmed founders were backcrossed to wild type (C57BL/6 J) mice, and the line was established from F1 heterozygous offspring carrying the confirmed allele. Experiments described were approved by the Animal Care Committee of IRCM (protocol 2021–09 YK). Primer used to genotype *Eya2* mice are: Eya2-fw: AAT​CTG​GCT​GCA​CTG​AAG​ATG​G; Eya2-rev: CTG​TAG​GAG​CAA​AGG​CCA​CTC.

### Skeletal preparation

Limbs were dissected and put overnight in ethanol 95%, then stained with Alcian blue for 24h, rinsed with ethanol 95% for 24h, dissolved in KOH 1% for 2 h and stained with Alizarin red for a few hours. Limbs were finally dehydrated in a series of ethanol: glycerol: water solutions and kept in a final solution of ethanol: glycerol (1:1). For the size comparison between controls and mutants, the autopod size was measured from the basal part of the proximal-most carpals bone to the tip of the third digit of flatten limbs. Statistical significance was assessed by two-tailed Welch’s unequal t-test. The number of limbs used for analysis is indicated in the figure legends.

### Whole-mount ISH

Whole mount *in situ* hybridization was done as previously described (Scotti and Kmita, 2012). Briefly, collected embryos were fixed overnight in 4% PFA, then dehydrated in a graded series of methanol: PBST and kept in 100% methanol at −20 °C. For the experiment, embryos were rehydrated, bleached in 6% hydrogen peroxide for 1 h on ice, treated with proteinase K (15min for E12.5 and 17min for E13.5 embryos), post fixed with 4% PFA for 20min, and probe hybridization was done overnight at 68 °C in hybridization buffer. Embryos were then washed in formamide-based solutions and incubated overnight with anti-digoxigenin antibody (Sigma Cat# 11093274910, diluted 1:3,000). Embryos were then washed several times in TBST, and the signal was revealed in the dark with NBT-BCIP. Finally, embryos were washed in PBST and kept in 1% PFA: PBST solution. Probes were synthesized using linearized plasmids or PCR products by reverse transcription with T7 RNA polymerase enzyme (NEB, M0251 S) and nucleotides DIG-RNA labelling mix (Sigma, Cat# 11277073910) for 1 h at 37 °C. Anti-sense probe for *Bmp7* and *Ihh* were generated previously (Hill et al., 1989; Vortkamp et al., 1996). Probes for *Aldh1a2*, *Adamts9*, *Mafb*, *Dkk1*, *Adra2a*, *Foxc2* and *Eya2* were generated using cDNA from limb buds with primers indicated in Table 1.

**TABLE 1:** ISH probe sets. Reverse primers contain a T7 sequence for *in vitro* transcription.

Gene	Forward primer sequence	Reverse primer sequence
*Aldh1a2*	GCT​CAC​CAG​GGT​GTG​TTC​TT	TAA​TAC​GAC​TCA​CTA​TAG​GGG​AGA​AAC​GTG​GCA​GTC​TT
*Adamts9*	TCT​GAG​TGC​TCA​GTG​ACC​TG	TAA​TAC​GAC​TCA​CTA​TAG​GGC​GGT​GGC​AAA​AGG​AAC​AG
*Mafb*	ATG​CAG​TTC​CCA​AAC​TCC​TGC	TAA​TAC​GAC​TCA​CTA​TAG​GGC​TGT​TTG​GCT​CAA​TGG​GAG
*Dkk1*	TTC​TCT​ATG​AGG​GCG​GGA​AC	TAA​TAC​GAC​TCA​CTA​TAG​GTT​AGT​GTC​TCT​GGC​AGG​TG
*Adra2a*	AAC​CGG​GAG​AAA​CGC​TTC​AC	TAA​TAC​GAC​TCA​CTA​TAG​GGT​TCA​TTA​ACC​CTT​TGG​CAG​C
*Foxc2*	GGA​ACC​TTC​TTC​GAC​GGA​GC	TAA​TAC​GAC​TCA​CTA​TAG​GTG​GCA​TCT​ACT​GTA​CAA​AGC​C
*Eya2*	CAT​GCC​TTT​CTG​GAG​GAT​ATC​CTG	TAA​TAC​GAC​TCA​CTA​TAG​GGC​CTA​AAC​AAC​GGG​CAA​AGG

### HCR staining

Hybridization Chain Reaction (HCR) was performed as described previously described (Morabito et al., 2023). Briefly, collected embryos were fixed overnight in 4% PFA, then dehydrated in a graded series of methanol: PBST and kept in 100% methanol at −20 °C. For the experiment, embryonic forelimbs were dissected and rehydrated, photobleached two times 45min, incubated in a detergent solution for 2h, and incubated overnight at 37 °C with the HCR probes in probe hybridization buffer. Limbs were then washed in probe wash buffer, equilibrated in the amplification buffer for several hours and incubated overnight at 24 °C with the hairpins. Limbs were then washed in SSCT solution with DAPI for 3h, washed in PBS and cleared overnight in a Fructose: Glycerol solution before confocal imaging (Leica SP8 confocal microscope). Probe details for *Col2a1*, *Col10a1*, *Rara* and *Rarg* are indicated in Table 2. For the digit size comparison between controls and mutants, digits were measured from the basal part of the metacarpals, at the junction with the signal from the future carpal bones, to the digit tip. Statistical significance was assessed by two-tailed Welch’s unequal t-test. The number of limbs used for analysis is indicated in the figure legends.

**TABLE 2:** HCR probe sets.

Gene	Accession number	Amplifier	Probe set size
*Col2a1*	NM_031163.3	B2 or B3	20
*Col10a1*	NM_009925	B2	20
*Rara*	NM_009024.2	B1	20
*Rarg*	NM_011244.5	B3	20
*Sox9*	NM_011448.4	B5	20
*Aldh1a2*	BC_075704.1	B3	20

### Nuclear protein extraction and western blot

Nuclear proteins were extracted from the distal part of E13.5 embryonic forelimbs of WT and *Eya2* ^*−/−*^ embryos. Briefly, limbs were smashed and resuspended in 400 µL of buffer A (10 mM HEPES pH7.9, 10mM KCl, 0.1 mM EDTA, 0.1 mM EGTA, 1 mM DTT, supplemented with PMSF 0.5 mM final and protease inhibitor cocktail) for 15 min on ice. Then, 50 µL NP40 10% was added and vortexed for 10 secondes. The mixture was then centrifuged, and the pellet was resuspended in 30 µL of Buffer C (20 mM HEPES pH7.9, 400mM KCl, 1 mM EDTA, 1 mM EGTA, 1 mM DTT, supplemented with PMSF 0.5 mM final and protease inhibitor cocktail) and vortexed for 30 min at 4 °C. After centrifugation, the supernatant was kept and dose with Bradford. The protein extraction was kept at −80 °C with Laemlli buffer and ß-mercaptoethanol. For the Western blot, 30 µg of proteins were loaded on an acrylamide gel and transferred to a PVDF membrane according to standard procedures. The membrane was incubated overnight at 4 °C with Eya2 antibody 1:500 (Proteintech 11314-1-AP) and Lamin-B1 antibody 1:1,000 (abcam ab16048) and then with a mouse anti-rabbit IgG-HRP 1:5,000 (Santa-Cruz sc2357) 1 h at RT. The signal was revealed with ECL solution and imaged with ChemiDoc Imaging System (Bio-Rad). The image presented in Supplementary Figure S4 is a representative result from two independent experiments.

### Lysotracker

Lysotracker was performed on embryonic forelimbs by incubating in Lysotracker Red (Invitrogen L7528, 5 µm final concentration) diluted in HBSS solution for 45 min at 37 °C. Limbs were then rinsed in HBSS and fixed overnight in 4% PFA. Limbs were then washed in PBS and dehydrated with solution of increasing concentration of methanol and then cleared in benzyl alcohol: benzyl benzoate (BaBB) solution. Images were taken with a Leica M165 FC fluorescent steromicroscope equipped with a Leica DFC450 C camera (for *Aldh1a2* control and mutant limbs) and Leica SP8 confocal microscope (for *Eya2* limbs).

### ROS detection

To determine ROS levels, embryonic forelimbs were dissected and incubated in 10 µM dihydroethidium (DHE–Cayman Chemical) in PBS (pH 6.5) 45 min at 37 °C. Limbs were then rinsed in PBS and fixed overnight in 4% PFA, then dehydrated with solution of increasing concentration of methanol and then cleared in BaBB solution and imaged with a Leica SP8 confocal microscope.

### TUNEL staining, immunostaining

Cryosections for TUNEL and immunostaining were made by fixing the embryos overnight in 4% PFA at 4 °C, then put in a 30% Sucrose solution, embedded in 30% Sucrose/OCT (1:1) and sectioned at 12 µm. TUNEL staining was performed with the Promega kit following manufacturer instructions and imaged under a DM6 fluorescent microscope. For immunostainings, sections were blocked 1 h in BSA 1%, Triton X-100 0.4%, and incubated overnight with Pecam/CD31 1:100 (BD 550274) or MSX1 1:200 (RD AF5045) and Ki67 1:300 (Thermofisher SP6 clone RM9106) antibodies, then incubated 45 min with secondary antibody (Alexa fluor 647 chicken anti-rat, Thermofisher, A21472 diluted 1:1,000 for Pecam; Cy5-conjugated goat anti-rabbit, Jackson ImmunoResearch, 111–176-003 diluted 1:500 for Ki67; Alexa fluor 568 donkey anti-goat, Invitrogen, A11057 diluted 1:500 for MSX1) and mounted in a DAPI mounting medium. Sections were imaged with a Leica SP8 confocal microscope.

Image analysis for the measurement of the vasculature area was done on ImageJ as previously described (Eshkar-Oren et al., 2015) and represented as the percentage of positive area relative to the area of the autopod. A total of at least 20 forelimb sections from three embryos for each genotype were used for the analysis.

For the quantification of cell proliferation, Ki67-positive cells were automatically counted in the different interdigital areas using the Analyze Particles on ImageJ. Briefly, interdigital domains were defined using MSX1 staining, and the threshold was adjusted on each image to extract the specific signal from Ki67 channel. Ki67-positive cells with a size >20 µm^2^ were then recorded using the Analyze Particles tool. The results in Supplementary Figure S3C are calculated as follow (number of Ki67+ cells/interdigital area [µm^2^]) x 100,000. A total of at least 20 forelimb sections from three embryos for each genotype were used for the analysis. For both Pecam area comparison and cell proliferation, the statistical difference between controls and mutants was determined by two-tailed Welch’s unequal t-test.

### RNA extraction and sequencing

The distal part, corresponding to the expression domain of *Hoxa13*, of embryonic limbs of individual embryos was dissected and stored at −80 °C until the day of experiment. RNA extraction was performed with the Qiagen RNeasy Plus Mini kit from three *Hoxa13* ^*Cre/+*^ embryos (six limb buds) controls and four *Aldh1a2* ^*c/c*^ mutants (eight limb buds) for E12.5 and three control and three mutant embryos for E13.5. Each replicate corresponds to the two distal forelimb dissected from one embryo. mRNA enrichment, library preparation and flow-cell preparation for sequencing were performed by the IRCM Molecular Biology Core Facility according to Illumina’s recommendations. Sequencing was done on an Illumina NovaSeq instrument with a 100-cycle paired-end configuration.

### RNA-seq analysis

The Galaxy online software (UseGalaxy.ca) was used for the RNA-seq analysis. First, FastQC v.0.74 was used to ensure for the correct quality of the fastq files. Then, paired-end reads were aligned to the mm10 reference genome with STAR v.2.7.11 b using default parameters. For measurement of gene expression, reads were counted from the output Bam files generated by STAR with featureCount v.2.1.1 with default parameters and strand information = Stranded (Reverse). Finally, DeSeq2 v.2.11.40.8 was used with default parameters on the output Count files from featureCount to perform the differential analysis. Statistical significance was assessed using the Wald test, and p-values were adjusted for multiple testing using the Benjamini–Hochberg false discovery rate (FDR) procedure. Genes with an adjusted p-value <0.05 were considered significant and were used for subsequent analysis.

### Chromatin immunoprecipitation (ChIP) and sequencing

Chromatin immunoprecipitation for H3K27ac was performed as previously described (Gentile et al., 2019) with few changes. The distal part of E13.5 embryonic limbs of individual embryos were dissected and cross-linked using 1% formaldehyde for 13 min at room temperature and stored at −80 °C until genotyping was performed. For each replicate (2 controls and two mutants), about 14 limbs were used. The chromatin was sonicated with a Covaris E220 and then kept overnight at −20 °C while quantifying an input of each replicate. The next day, Protein A and Protein G Dynabeads (Invitrogen) were incubated for 6 h at 4 °C with 3 µg H3K27Ac antibody (CST – 8173 S). After input quantification, 15 µg of chromatin was coupled to the beads overnight at 4 °C. Finally, the beads were washed in low salt (1% Triton, 0.1% SDS, 150 mM NaCl, 20 mM Tris (pH8), 2 mM EDTA), high salt (1% Triton, 0.1% SDS, 500 mM NaCl, 20 mM Tris pH8, 2 mM EDTA), LiCl (1% NP-40, 250 mM LiCl, 10 mM Tris (pH8), 1 mM EDTA) and TE buffer (50 mM NaCl, 10 mM Tris (pH8), 1 mM EDTA). Lastly, after elution for 6 h at 65 °C (in 1% SDS, 50 mM Tris (pH8), 10 mM EDTA), the chromatin was treated with RNaseA (BioBasic) for 15 min at RT, followed by Proteinase K treatment for 1 h at 55 °C. The DNA was then purified on QIAquick columns (Qiagen). Sequencing was performed on an Illumina NovaSeq instrument with a 100-cycle paired-end configuration.

### ChIP-seq analysis

HOXA13 and HOXD13 ChIP-seq data shown in Figure 2 were previously published (Sheth et al., 2016), accession number GSM2151013 and GSM2151014.

**FIGURE 2:**
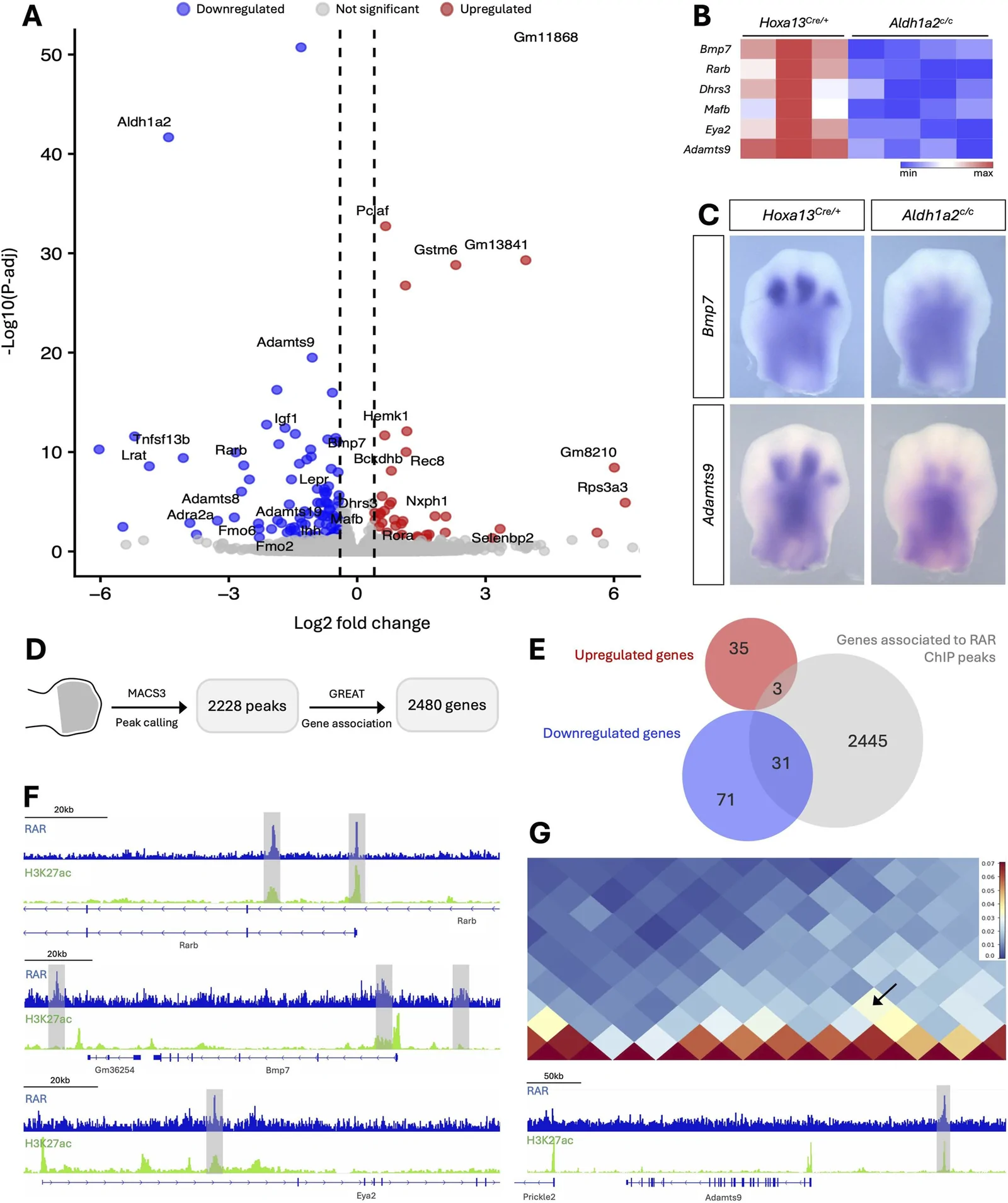
Transcriptome analysis of *Aldh1a2* ^*c/c*^ distal limb buds at E12.5. **(A)** Volcano plot showing the differentially expressed genes in the distal forelimb at E12.5 between *Hoxa13* ^*Cre/+*^ control embryos (n = 3) and *Aldh1a2* ^*c/c*^ mutants (n = 4). For each embryo, both forelimbs were used. Genes significantly downregulated and upregulated (p-value <0.05 and log2FC < −0.4 or log2FC > 0.4) are shown in light blue and red, respectively. Dashed lines indicate cutoffs of |log2FC| > 0.4. **(B)** Heatmap of RPKM expression of several known and newly identify RA targets. **(C)** Whole mount *In Situ* Hybridization showing the expression of *Bmp7* and *Adamts9* in the forelimb of *Hoxa13* ^*Cre/+*^ control and *Aldh1a2* ^*c/c*^ embryos at E12.5 (n = 3). **(D)** Association of the 2,228 significant RAR-bound sites (p < 10^−5^ from ChIP-seq peaks) from E11.5 distal forelimb to the surrounding genes identifies 2,480 potential direct targets (made with GREAT software). **(E)** Venn diagram showing the direct targets of RA/RAR, identified by crossing the list of up- and downregulated genes from the RNA-seq (with |log2FC| > 0.4) to the list of genes identified from the RAR ChIP peaks. The 31 putative direct targets downregulated are: *L1td1, Rbpj, Pxylp1, Mid1, Esrrb, Dhrs3, Cyb5r4, Mafb, Bmper, Plk2, Piezo2, Aff3, Rarb, Pitpnc1, Adamts9, Meioc, Bcl2, Ccdc3, Sned1, Ptch1, Igf1, Spsb4, Masp1, Fmo6, Car12, Fgfr2, Foxc1, Gprc5a, Eya2, Bmp7* and *Spsb1*. **(F)** IGV tracks showing examples of RAR and H3K27ac (E12.5 from Gentile et al., 2019) ChIP peaks at three direct targets: *Rarb* (top), *Bmp7* (middle) and *Eya2* (bottom). RAR significant peaks (p < 10^−5^) are highlighted in gray. **(G)** Hi-C data obtained from E12.5 distal limb buds (Rodriguez-Carballo et al., 2017) shows enriched contact (black arrow) between the promoter of *Adamts9* and a RAR-bound site (highlighted in gray) located more than 100 kb upstream.

RAR ChIP-seq data were recovered from published data (Yakushiji-Kaminatsui et al., 2018), accession number GSM2824388. The fastq files were aligned to the mm10 reference genome using bowtie2 v2.5.4. Blacklisted regions were filtered using bedtools v2.31.0, and samtools v1.20 was used to generate the Bam files. Duplicated reads were removed using picard v3.1.0. A bigwig file was generated for visualization on IGV software using Homer v.4.11 with the following parameters: norm 1,000,000 -o auto -bigWig -fsize 1e20. Significant RAR peaks were identified with macs3 callpeak function with the parameter–pvalue 0.00001.

H3K27ac ChIP-seq data were processed as described above for the RAR ChIP-seq. For visualization, bigwigs from both replicates in the two conditions were merged using deeptools command bigwigAverage with the parameter--binSize 25. Significant acetylation peaks were identified with macs3 callpeak function with the parameter–pvalue 0.00001. Peaks identify in both replicates of each condition were kept using the mergePeaks function of Homer v.4.11, and these peaks from the two conditions were pooled to get the total list of peaks used for the differential analysis. Differential analysis was performed using Homer v4.11. Raw read counts were quantified over the merged peak set using analyzeRepeats.pl with the -raw option. Differential enrichment analysis was then performed using getDiffExpression.pl, which uses DESeq2 to model read counts with a negative binomial generalized linear model. Statistical significance was assessed using the Wald test, and p-values were adjusted for multiple testing using the Benjamini–Hochberg false discovery rate (FDR) procedure. The input used for both RAR and H3K27ac peakcalling analysis with macs3 was previously published in Gentile et al., 2019, accession number GSM3508381.

GREAT (Genomic Regions Enrichment of Annotations Tool) software was used to annotate the ChIP-seq peaks (both from the RAR and H3K27ac) to the nearby genes. Species assembly mm10 was selected and bed files were provided as input. The analysis was performed with the Basal plus extension mode with default parameters.

### Hi-C data visualization

For visualization of the Hi-C data, the file GSE161378_E12_DL_40 kb.cool.gz was recovered from GEO with the accession number GSE161378 and imported on the Galaxy instance software as “cool” file. PyGenomeTracks tool was used on the Galaxy software (UseGalaxy.ca) with the following parameters: Region of the genome to plot: chr6:92, 680, 812–93,203,051 for Adamts9 and chr4:144, 679, 350–145,220,402 for Dhrs3; Minimum value: 0; Plot the transformed value: no transformation; Scale factor: 1,0; Choose style of the track: Gene track/Bed track; height: 10; Put all labels inside the plotted region: Yes; Bed style: UCSC.

## Results

### Aldh1a2 conditional mutant exhibit syndactyly due to the loss of interdigital apoptosis

To assess the function of *Aldh1a2*, and thus retinoic acid signaling, in the forelimb autopod, we used the *Hoxa13:Cre* deleter strain (Scotti et al., 2015) to conditionally inactivate *Aldh1a2* in the distal limb mesenchyme. This genetic approach allowed us to bypass the early lethality of *Aldh1a2* mutant and to investigate its phenotype at stages that could not be previously assessed when using RA supplementation to rescue the early lethality of *Aldh1a2 null* embryos (Niederreither et al., 1999). *Aldh1a2* inactivation in the distal limb bud was confirmed by *in situ* Hybridization Chain Reaction (HCR; Supplementary Figure S1A). *Hoxa13* ^*Cre/+*^ *; Aldh1a2* ^*flox/flox*^ (referred to as *Aldh1a2* ^*c/c*^ onwards) newborns display a severe syndactyly phenotype, with the tips of each digit being barely visible (Figure 1B). This syndactyly only involves soft tissue, as skeletal elements are properly separated (Figure 1C). This phenotype persists over time as illustrated by the limb phenotype of three-week-old mice (Supplementary Figure S1B) and is also observed in hindlimbs of *Aldh1a2* mutants, but not in *Hoxa13* ^*Cre/+*^ or *Aldh1a2* ^*flox/flox*^ controls (Supplementary Figures S1C,D). The absence of interdigital tissue regression is already visible at embryonic stages. Indeed, at E13.5, mutant embryos show impaired tissue regression in the interdigital area (black arrowheads, Figure 1D upper panel). The resulting syndactyly is much more pronounced at E14.5, a stage at which digits are normally fully separated in WT embryos while they are completely fused in *Aldh1a2* mutants (Figure 1D–bottom panel). Lysotracker staining, which detects acidic compartments in cells, together with TUNEL and cleaved Caspase-3 stainings, show that cell death is almost completely abolished in the interdigital area (Figure 1E; Supplementary Figure S1E).

### Transcriptomic analysis identifies new retinoic-acid targets

To date, very few genes are known as having a role in interdigital tissue regression and being regulated by RA in the distal limb, namely, *Bmp7* and *Rarb.* However, even though Bmp signaling is important for interdigital tissue regression (Guha et al., 2002), *Bmp7* mutant phenotype is relatively mild (Kaltcheva et al., 2016) compared to that of *Aldh1a2* ^*c/c*^. Moreover, *Rarb* inactivation leads to interdigital webbing only when combined with *Rarg* inactivation (Ghyselinck et al., 1997). The RA target *Mmp11* was also proposed to contribute to interdigital tissue regression based on its downregulation upon impaired RA signaling (Dupé et al., 1999; Zhao et al., 2010) but its inactivation has no effect on interdigital tissue regression (Fanjul-Fernández et al., 2010 and MGI database). Together, these results suggest that other RA targets are implicated in interdigital tissue regression. To identify new targets involved in this process, we performed a comparative transcriptomic analysis using the distal part of E12.5 embryonic forelimbs. Given that the Cre recombinase is a knock-in at the endogenous *Hoxa13* gene (Scotti et al., 2015), *Hoxa13* ^*Cre/+*^ genotype was used as control. We chose E12.5 for our transcriptomic analysis based on the temporal specificity of the *Hoxa13* ^*Cre*^ allele*.* Indeed, it was previously shown that *Hoxa13* ^*Cre/+*^ -mediated mRNA clearing of a floxed allele begins at E11.25 in the posterior distal tip of the limb bud and is fully achieved by E11.75 while the complete clearing of the corresponding protein is expected to occur slightly later depending on the half-life of the protein (Lopez-Rios et al., 2012). As we performed bulk RNA-seq from distal limb buds, deregulated genes are not specific to the interdigit tissue and can include gene mis-regulation in other part of the limb bud such as digit condensations, ectoderm or blood vessels. Among the downregulated genes, we identified known RA targets, such as *Bmp7* (Dupé et al., 1999; Rodriguez-Leon et al., 1999), *Rarb* (Berenguer et al., 2020; Paschaki et al., 2013; Zhao et al., 2010), *Dhrs3* (Berenguer et al., 2020; Paschaki et al., 2013), *Eya2* (Ali-Khan and Hales, 2006) and *Mafb* (Figures 2A-B; Supplementary Table S1A,C). The latter was shown to respond to RA in chick limbs through bead implantation experiment (Suda et al., 2014). Similarly, *Eya2* was identified as being downstream of RA signaling as it is upregulated in limbs cultured in the presence of teratogenic concentration of vitamin A (Ali-Khan and Hales, 2006). Our data show that these genes are indeed regulated by physiological concentrations of RA in mouse limbs (Figures 2A,B). Importantly, our transcriptomic analysis allowed us to identify new RA targets relevant to the syndactyly phenotype. This includes several Adamts enzymes, which are metalloproteinases of the extracellular matrix, among which *Adamts9* was the most significantly downregulated. This was confirmed by *in situ* hybridization where a clear decrease in expression is visible in the interdigital tissue (Figure 2C). Interestingly, *Adamts9* is involved in the process of interdigital apoptosis in mice, as its conditional knock-out in limbs leads to a syndactyly phenotype, though relatively mild (Dubail et al., 2014). Other factors known to be involved in the process of interdigital apoptosis, like *Bmp2* (Kaltcheva et al., 2016) and *Fgf8* (Hernandez-Martinez et al., 2009; Pajni-Underwood et al., 2007), were not affected at this stage in *Aldh1a2* ^*c/c*^ limb buds (Supplementary Figure S2A).

**FIGURE 3:**
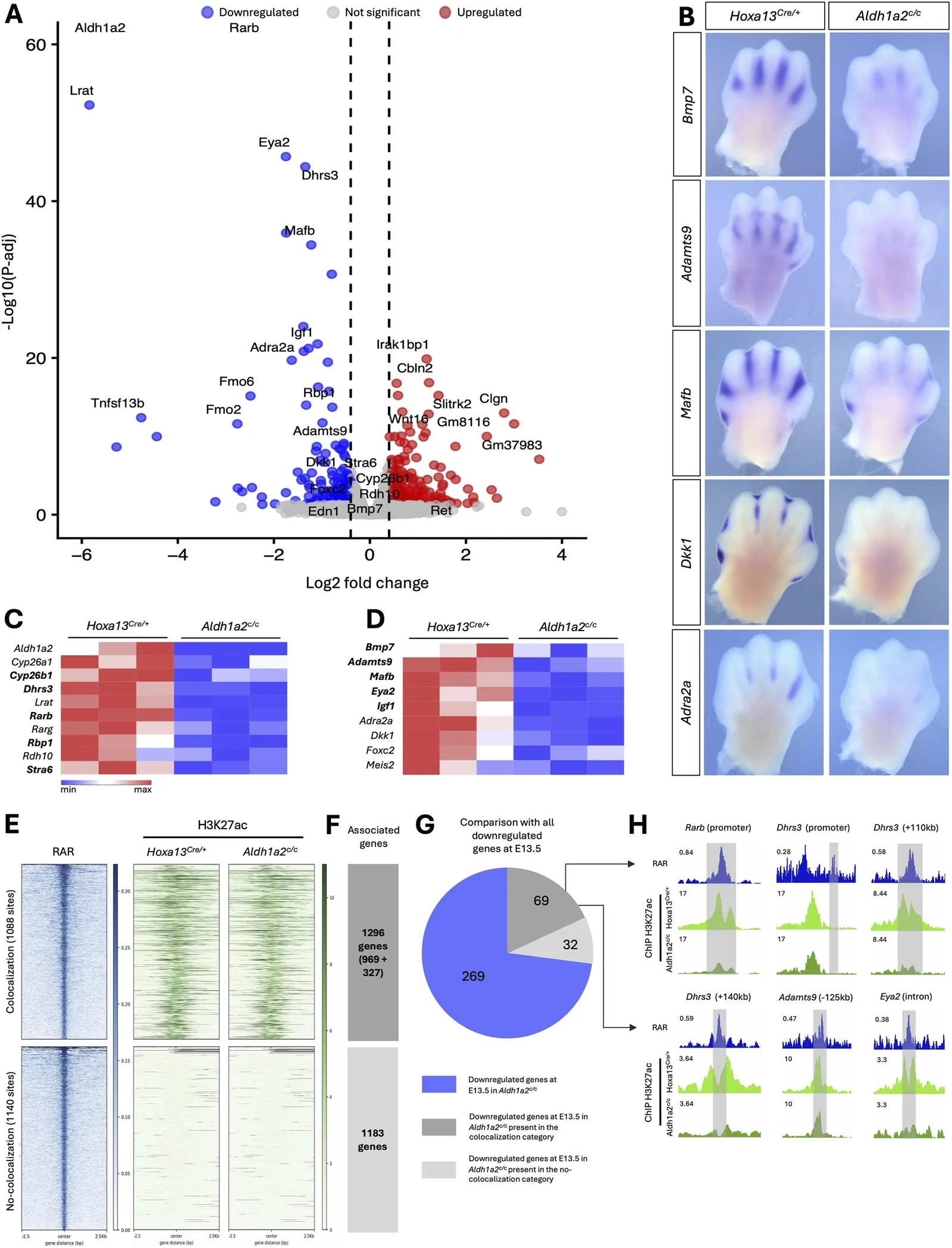
Transcriptome analysis of *Aldh1a2* ^*c/c*^ distal limb buds at E13.5. **(A)** Volcano plot showing the differentially expressed genes at E13.5 between *Hoxa13* ^*Cre/+*^ controls (n = 3) and *Aldh1a2* ^*c/c*^ embryos (n = 3). Genes significantly downregulated and upregulated (p-value <0.05 and log2FC < −0.4 or log2FC > 0.4) are shown in blue and red, respectively. **(B)** Whole mount *In Situ* Hybridization showing the expression of *Bmp7*, *Adamts9*, *Mafb*, *Dkk1* and *Adra2a* in the forelimb of *Hoxa13* ^*Cre/+*^ control and *Aldh1a2* ^*c/c*^ limb buds at E13.5 (n = 3 except for *Adra2 in situ* hybridization for which n = 2). **(C,D)** Heatmap showing the expression (RPKM normalized) of genes related to RA metabolism **(C)** and of genes related to interdigital apoptosis **(D)** in *Hoxa13* ^*Cre/+*^ control and *Aldh1a2* ^*c/c*^ mutant distal limbs at E13.5. The genes highlighted in black are putative direct targets with a RAR binding sites in the vicinity of the gene. **(E)** Heatmaps showing all the significant RAR-bound sites (2,228) and H3K27ac in *Hoxa13* ^*Cre/+*^ control and *Aldh1a2* ^*c/c*^ mutant distal forelimbs at E13.5. The colocalization category (1,088 regions) corresponds to loci where RAR binding colocalizes with H3K27ac signal in control limbs. The no-colocalization category (1,140 regions) corresponds to loci where no H3K27ac is present at RAR-bound sites in control limbs. The signal for H3K27ac is shown as the average from two replicates for each condition, and regions are ranked according to the signal from the RAR ChIP. **(F)** GREAT association of genes from the two categories of RAR binding. The 1,296 genes from the colocalization category comprise genes that harbor a RAR sites that is acetylated in controls forelimbs (969 genes), but also genes that are found in both categories (meaning that they have at least one RAR sites that is acetylated) (327 genes). The 1,183 genes from the no-colocalization category comprise genes associated with a RAR-bound site that is not acetylated in controls forelimbs **(G)** Comparison of the genes associated with the two categories presented in **(F)** with all the significantly downregulated genes at E13.5 (370 genes). The 69 downregulated genes associated with the colocalization category include: *Tmem30b, Twist2, Adamts9, Hadh, Rarg, Tbx3, Gse1, Aqp4, Smyd2, Rusc1, Smad6, Fbln1, Msx1, Sned1, Ccdc68, Art4, Npl, Homer3, Gdf5, Pfn1, Gpx4, Mylk, Phyhip, Cyp26b1, Pik3r1, Mrpl34, Hes1, Stra6, Pxylp1, Twist1, Spsb4, Snai1, Hic1, Eef1b2, Klf3, Gpi1, Ncoa3, Aebp2, Ndufs8, Car12, Gsta4, Fgfr2, Babam2, Rbp1, Tbx2, Eif2s2, Parp16, Dhrs3, Id3, Cyp26a1, Calml4, Irx6, Bid, Eya2, Cox5b, Stx18, Bmp7, Spsb1, Ezr, Sncaip, Plk2, Nxn, Pbx1, Rarb, Foxo1, Ndufs6, Hdhd3, Pitpnc1* and *Ptch2.* The 32 downregulated genes associated with the no-colocalization category include: *Batf3, Add3, Ust, Ccdc3, Rasl10b, Pth1r, Cox7b, Vsir, Ankrd44, F11r, Hlx, Meis2, Grb14, Igf1, D030056L22Rik, Abcc4, Grik4, Masp1, Fmo6, Mroh1, Slc8a1, Nsg2, Pax1, Sp7, Pthlh, Aifm2, Gprc5a, Rasl11a, Mafb, Sst, Egln3* and *Zfp536*. **(H)** IGV tracks showing loci with a significant decrease (p-adj <0.05) in H3K27ac (top), and other regions with a visible decrease in H3K27ac significant with (p-value <0.01) (bottom). RAR significant peaks (p < 10^−5^) are highlighted in gray.

We next wondered whether the differentially expressed genes are directly regulated by RA, i. e., whether RAR-bound sites exist in the vicinity of these genes. To address this question, we used publicly available ChIP-seq data of the RARs that was performed in the distal part of E11.5 embryonic limbs (Yakushiji-Kaminatsui et al., 2018). We identified a total of 2,228 significant RAR peaks (p < 10^−5^), which were then associated to nearby genes with GREAT (Genomic Regions Enrichment of Annotations Tool, up to 1 Mb from the peak). This led to the identification of 2,480 genes that are putative direct targets of the RARs (Figure 2D). We next crossed the list of differentially expressed genes (with a |log2FC| > 0.4) with the set of genes identify with GREAT. While our RNA-seq was done at E12.5, the RAR ChIP-seq in the limb bud was done at E11.5 (Yakushiji-Kaminatsui et al., 2018), it is thus possible that there are variations in genome wide binding of RARs at E12.5 compared to E11.5 and therefore this association between RAR binding and deregulated genes only inform us on putative direct target genes. We found 34 genes with altered expression in *Aldh1a2* ^*c/c*^ and having RAR target site nearby (Figures 2E). This list of 34 genes contains most of the genes related to apoptosis including *Rarb*, *Bmp7, Mafb, Eya2* and *Adamts9*. For some of them, such as *Rarb, Bmp7* and *Eya2,* RARs are bound in the gene body or in close vicinity of the promoter (Figure 2F). For other targets, like *Adamts9* or *Dhrs3*, the RAR bound sites are located more distantly. To test whether the latter RAR targets could be enhancer elements for *Adamts9* and *Dhrs3* respectively, we took advantage of publicly available Hi-C data from distal embryonic E12.5 limbs. Hi-C data analysis shows enriched contacts between the RAR targets and the gene promoter, thus supporting that *Adamts9* and *Dhrs3* are likely RA-direct targets (Figure 2G; Supplementary Figure S2C).

Altogether, these results show that *Aldh1a2* (and thus RA signaling) regulates the expression of several genes related to apoptosis and important for interdigital tissue regression, some of which were previously identified in different systems and/or under non-physiological conditions, and identify *Adamts9* as a new RA target contributing to interdigital tissue regression.

### Transcriptomic at E13.5 identifies the full set of RA targets required for interdigital tissue regression

Even though we identified new RA targets downregulated in *Aldh1a2* ^*c/c*^ just before the appearance of interdigital webbing, we decided to perform another transcriptomic analysis at a later stage to get a complete assessment of the transcriptional changes resulting from the loss of RA signaling. We thus performed RNA-seq using the presumptive autopod domain of E13.5 embryos. As anticipated, we identified more DEGs (Figure 3A–151 up and 99 down with a |log2FC| > 0.585; 258 up and 167 down with a |log2FC| > 0.4; Supplementary Table S1B,D), approximately 20% of them being potential direct targets (Supplementary Figure S3B). Among the DEGs, many downregulated genes are related to RA metabolism (Figure 3C), including *Dhrs3* and *Lrat*, which act as negative regulators of RA, the RA-degrading enzymes *Cyp26a1* and *Cyp26b1* but also the RARs beta and gamma as well as *Rbp1* and *Stra6*, which promotes RA signaling. The latter is known to be a direct target of RA (Bouillet et al., 1997). This shows that the feedback mechanism that exists in other systems to control RA homeostasis (O'Connor, Varshosaz and Moise, 2022) also takes place in the developing limb. As both positive and negative regulators of RA signaling are downregulated upon *Aldh1a2* inactivation, it is possible that RA signaling control these genes with opposite function in distinct compartment of the limb bud (digital versus interdigital domains). However, we cannot assess this possibility based on our bulk RNA-seq data. Downregulated genes also comprise those identified at E12.5 including *Bmp7, Adamts9, Mafb, Eya2* and *Adra2a* (Figures 3A,B,D). Among the genes only deregulated at E13.5, we identified *Dkk1*, a Wnt inhibitor that has a negative feedback effect to limit Wnt signaling (Zorn, 2001). Interestingly, *Dkk1* expression in the distal limb is restricted to the interdigital area and *Dkk1* mutant display a syndactyly phenotype (Grotewold and Rüther, 2002; Mukhopadhyay et al., 2001). As for *Bmp7, Adamts9, Mafb* and *Adra2a*, whole mount *in situ* hybridization shows that *Dkk1* expression is strongly downregulated in the interdigital tissue of *Aldh1a2* mutants (Figure 3E). However, *Dkk1* downregulation was not associated with an increased cell proliferation in the interdigital domains (Supplementary Figure S3C), suggesting that the effect of *Dkk1* misregulation is unrelated to Wnt-mediated proliferation in this context. Besides *Bmp7, Dkk1, Mafb, Adra2* and *Adamts9*, the other genes that have been previously implicated in interdigital apoptosis are not deregulated in the *Aldh1a2* mutant or have a log2 fold change smaller than 0.4 (Supplementary Figure S3D). Similarly, components of the extrinsic and intrinsic apoptosis pathway, were not transcriptionally affected in *Aldh1a2* mutant (Supplementary Figure S3E), except *Bid* and *Bax* which show a modest downregulation (log2 fold change = −0.42 and −0.24, respectively), the latter having already been shown to be responsive to RA in the distal limb (Hernandez-Martinez et al., 2009). Together, these results indicate that for proper interdigital tissue regression to occur, RA signaling acts on the degradation of the extracellular matrix, through *Adamts9*, and positively regulates the expression of other factors related to apoptosis, namely, *Bmp7*, *Dkk1*, *Mafb* and *Adra2a*.

**FIGURE 4:**
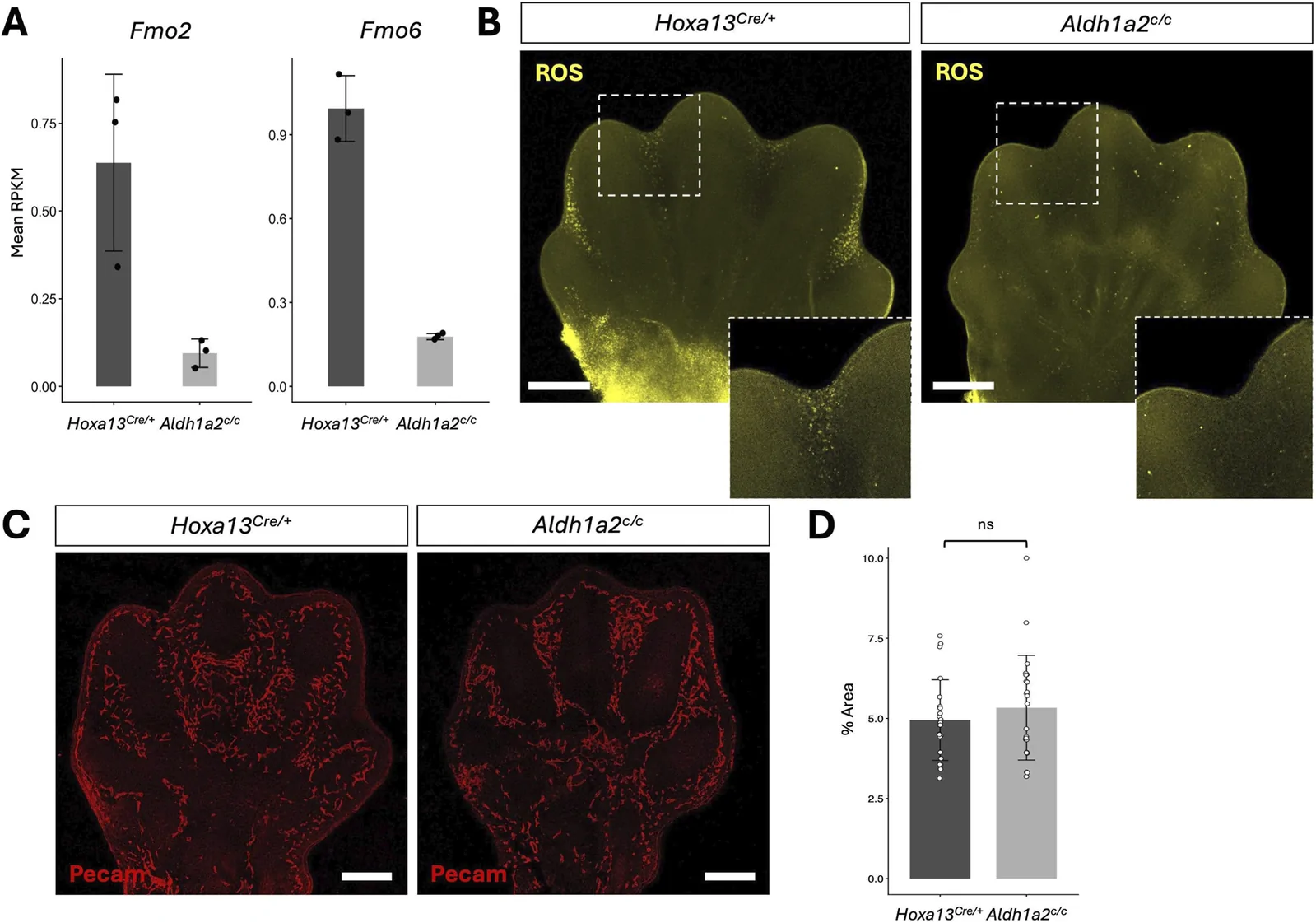
Loss of ROS in the interdigital tissue of *Aldh1a2* ^*c/c*^ limb buds. **(A)** Expression levels (RPKM normalized) of *Fmo2* and *Fmo6* from the RNA-seq data from *Hoxa13* ^*Cre/+*^ control and *Aldh1a2* ^*c/c*^ mutant distal limb buds at E13.5. **(B)** Reactive oxygen species (ROS) staining on *Hoxa13* ^*Cre/+*^ control and *Aldh1a2* ^*c/c*^ mutant forelimbs at E13.5 (n = 3). Scale bar is 250 µm. **(C)** Pecam immunostaining on cryosections from *Hoxa13* ^*Cre/+*^ control and *Aldh1a2* ^*c/c*^ mutant forelimbs at E13.5. Scale bar is 250 µm. **(D)** Quantification of the Pecam-positive area in the autopod domain of *Hoxa13* ^*Cre/+*^ control and *Aldh1a2* ^*c/c*^ mutant embryos. Statistical difference was determined by two-tailed Welch’s unequal variance t-test. Ns: not significant.

We then performed ChIP-seq for H3K27ac, which marks transcriptionally active regions, using E13.5 control limb buds to establish which RAR targets are transcriptionally active. Approximately 50% (1,088/2,228) of the RAR-bound loci at E11.5 colocalized with a significant H3K27ac peak in E13.5 distal forelimb tissue (Figure 3E), suggesting that the other 50% are either repressed regulatory elements or are not bound by RARs at E13.5. Comparison with the list of downregulated genes at E13.5 reveals 69 downregulated genes neighboring a RAR target decorated with the active histone mark, H3K27ac, in control limb buds (Figures 3F,G).

Then, to identify the effect of RA loss on the activity of regulatory elements bound by the RAR, we compared H3K27ac ChIP-seq in *Aldh1a2* ^c/c^ and control limb buds. Surprisingly, when performing the differential analysis between control and mutant samples using an adjusted p-value <0.05, decreased H3K27ac is significant only at few RAR targets (Supplementary Table S1E; Supplementary Table S2). These include *Rarb* and *Dhrs3* promoters, and a regulatory region of *Dhrs3* bound by RAR (Figure 3H–top; Supplementary Figure S2C). We repeated the analysis using a p-value <0.01 and with this parameter, a clear decrease in H3K27ac in *Aldh1a2* mutant is observed at RAR targets associated with genes related to apoptosis (Supplementary Figure S3F). This includes other regulatory regions of *Dhrs3* and *Rarb*, an upstream element at the *Adamts9* locus, and an intronic region of *Eya2* (Figure 3H–bottom; Figure 2G; Supplementary Figure S2C). Overall, these results show that the activity of only few RA target regulatory elements are affected by the loss of RA in the distal limb, but the ones that show a decrease in H3K27ac in *Aldh1a2* mutants are associated with downregulated genes relevant to the loss of apoptosis.

### Eya2 is not involved in the process of interdigital apoptosis

Among the candidate RA target genes identified in our transcriptomic and ChIP-seq analyses, we focused on *Eya2* because it was strongly downregulated in *Aldh1a2* mutants at both E12.5 and E13.5 and it is expressed in the interdigital tissue (Xu et al., 1997). Consistent with the transcriptomic data, whole mount *in situ* hybridization confirmed a marked loss of *Eya2* expression in mutant forelimbs compared with controls (Supplementary Figure S4A). In addition, *Eya2* is associated with a RAR target regulatory element showing reduced H3K27ac upon loss of RA signaling and *Eya2* has been reported to promote apoptosis in murine myeloid cells (Clark, Fee and Cleveland, 2002). These observations raised the possibility that *Eya2* mediates, at least in part, RA-dependent interdigital apoptosis. To test this hypothesis, we generated a loss-of-function mutation by deleting the fourth exon, which is common to all the different reported transcripts, leading to a frameshift that creates many downstream stop codons (Supplementary Figure S4B). The deletion of exon four was confirmed by PCR genotyping (Supplementary Figure S4C) and the absence of EYA2 protein was validated by Western blot (Supplementary Figure S4D). Morphological analysis of 2 weeks old mutant pups does not show any webbing phenotype (Supplementary Figure S4E). Consistent with this lack of effect on interdigital tissue regression, lysotracker staining at E13.5 in the *Eya2* mutant is similar to that of wild type limb buds (Supplementary Figure S4F). Together, these results indicate that *Eya2* does not play a role in the process of interdigital apoptosis.

### ROS are not generated in the interdigital tissue of Aldh1a2 mutants

Two of the top downregulated genes at E13.5 are *Fmo2* and *Fmo6* (Figure 3A and Figure 4A) and *Fmo2* is expressed in the interdigital tissue (GenePaint database; Visel, Thaller and Eichele, 2004). Flavin containing monooxygenases (Fmo) produce reactive oxygen species (ROS) (Siddens et al., 2014). Interestingly, in the developing limbs buds, ROS are present in the interdigital tissue and were shown to be involved in the process of interdigital apoptosis (Covarrubias et al., 2008; Salas-Vidal et al., 1998). We thus wonder whether ROS production was affected in *Aldh1a2* ^*c/c*^ limb buds. To address this question, we performed dihydroethidium (DHE) staining on E13.5 forelimbs to detect ROS. We observed an almost complete loss of DHE signal in the interdigital tissue of *Aldh1a2* mutant (Figure 4B). Interestingly, even though the vasculature was shown to contribute to the generation of ROS in the interdigital tissue (Eshkar-Oren et al., 2015), the loss of ROS in *Aldh1a2* ^*c/c*^ limb buds is not associated with detectable vascularization defect (Figures 4C, D). These results show that RA signaling also acts on the generation of ROS to induce apoptosis in the interdigital tissue, possibly through the downregulation of *Fmo2* and *Fmo6*.

**FIGURE 5:**
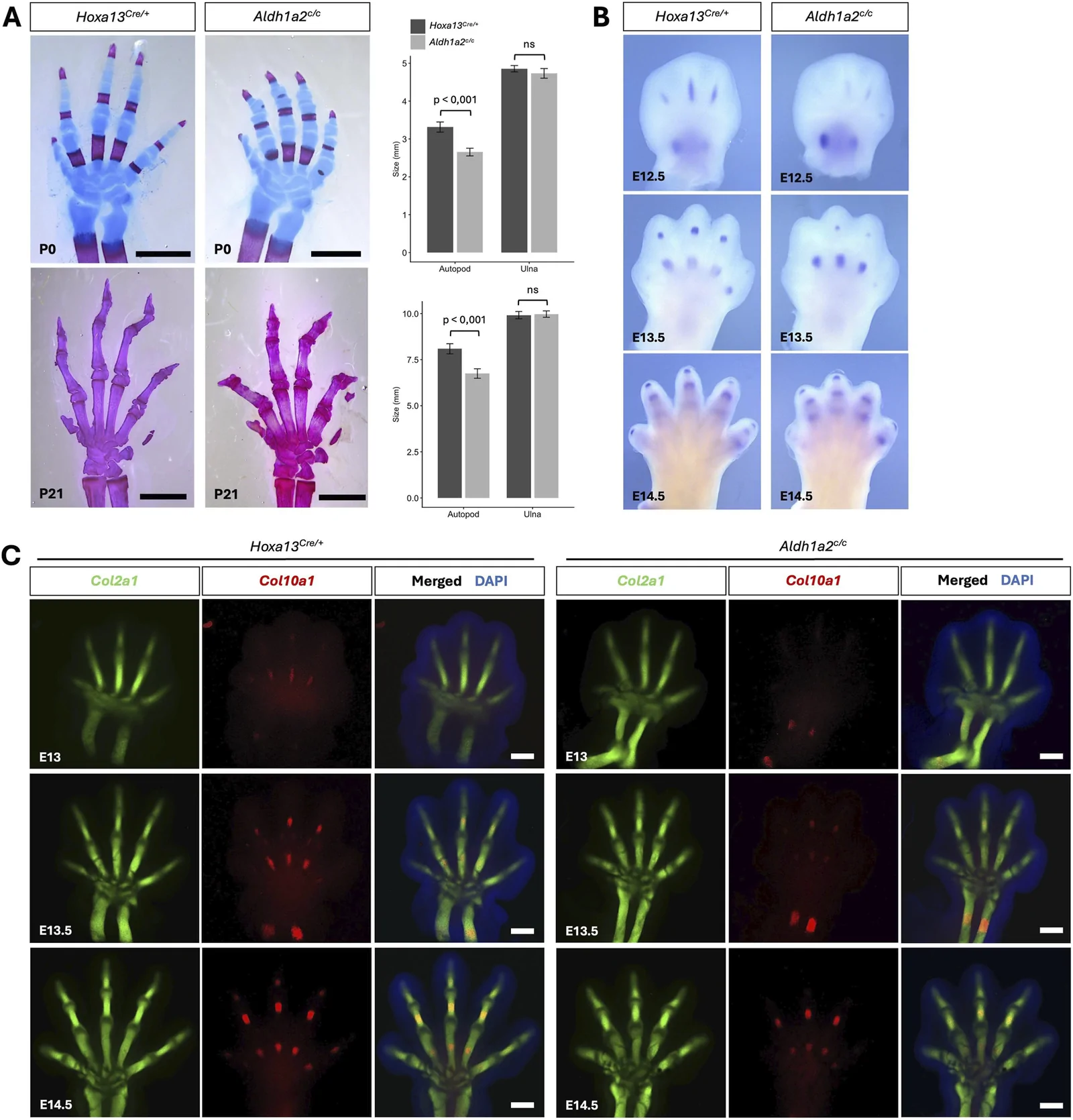
The conditional inactivation of *Aldh1a2* results in altered digit development. **(A)** Skeletal preparation of newborn (P0, top) and 3 weeks old (P21, bottom) *Hoxa13* ^*Cre/+*^ controls and *Aldh1a2* ^*c/c*^ mutant distal forelimb. Measurement of the autopod and of the ulna is shown on the right. P-values were determined by two-tailed Welch’s unequal variance t-test. N = number of limbs measured: control P0 n = 8, mutant P0 n = 6, control P21 n = 6, mutant P21 n = 6. Ns: not significant. Scale bar is 1 mm for P0 and 2 mm for P21. **(B)** Whole mount *In Situ* Hybridization of *Ihh* on *Hoxa13* ^*Cre/+*^ control and *Aldh1a2* ^*c/c*^ mutant forelimbs at E12.5, E13.5 and E14.5. **(C)** Hybridization Chain Reaction staining of *Col2a1* and *Col10a1* on *Hoxa13* ^*Cre/+*^ control and *Aldh1a2* ^*c/c*^ mutant forelimbs at E13, E13.5 and E14.5. Scale bar is 250 µm.

### RA effect on digit development

Even though the most striking phenotype visible at birth is the severe soft tissue syndactyly, *Aldh1a2* mutants also display mild skeletal defects (Figure 1C). Indeed, newborn mutants have a significantly smaller autopod compared to *Hoxa13* ^*Cre/+*^ controls, while the proximal bones of the limb are unaffected (Figure 5A – top). The smaller autopod size is not due to a developmental delay, as 3 weeks old individuals also exhibit the same size difference, along with exacerbated digit malformation (Figure 5A–bottom). Gene ontology performed on the downregulated genes from the RNA-seq at E12.5 and E13.5 revealed terms associated with bone and skeletal system development (Supplementary Figure S2B; Supplementary Figure S3A). This includes, for example, *Timp3, Lepr, Col2a1, Igf1* and *Ihh* at E12.5 and *Lepr, Edn1, Ogn, Igf1*, *Pthlh*, *Gdf5* and *Foxc2* at E13.5 (Supplementary Figure S5A). Given that such genes were downregulated as early as E12.5, we decided to check whether the digit condensations were already affected at this stage. We performed *in situ* Hybridization Chain Reaction (HCR) using *Col2a1* probe as an early chondrocyte marker to visualize digit formation. Measurements at either E12.5 or E13.5 did not reveal a significant difference in the length of the developing digits (Supplementary Figure S5B). It is only from E14.5 that a significant decrease in size started to be detected for each digit (Supplementary Figure S5B). We next performed HCR using a probe for *Sox9,* a key early regulator of chondrogenesis. No significant difference between the wild-type and the *Aldh1a2* mutant at E12.5 (Supplementary Figure S5C), consistent with the RNA-seq data showing no significant changes in *Sox9* expression neither at E12.5 nor at E13.5 (Supplementary Figure S5D). This suggests that the skeletal defect visible at birth is not due to defect in the early steps of chondrogenesis but occurs subsequently. *Ihh*, a marker of pre-hypertrophic and early hypertrophic chondrocytes, is important for chondrocyte proliferation and hypertrophy (Kozhemyakina, Lassar and Zelzer, 2015). This gene has an approximate 2-fold reduction at E12.5 in our RNA-seq dataset. We confirmed this downregulation using whole mount *in situ* hybridization (Figure 5B), which shows that while *Ihh* expression is visible in the metacarpals of control embryos, it is significantly reduced in the mutant autopod. At E13.5, *Ihh* is strongly expressed in the metacarpals of the mutant but, in contrast to the control, is barely detected in the first forming phalanx. This difference is again visible at E14.5, where a strong expression is detected at the tip of the digits in the control but to a lesser extent in the mutant digit tips (Figure 5B). This result shows that *Aldh1a2* inactivation leads to a delayed *Ihh* expression in the digit condensations. We also found a decreased expression of *Foxc2* in the hypertrophic zone of the latest forming digit segment in *Aldh1a2* mutant (Supplementary Figure S5E) and interestingly, *Foxc2* was shown to be important for chondrocyte proliferation and hypertrophy in the limb skeleton (Almubarak et al., 2021; Almubarak et al., 2024). Given that early chondrogenesis occurs normally and that genes important for chondrocyte proliferation and hypertrophy are downregulated (namely, *Ihh* and *Foxc2*), we decided to further investigate whether the process of chondrocytes differentiation was affected in *Aldh1a2* mutant. We thus performed HCR using a probe against *Col10a1*, a marker of early hypertrophic chondrocytes (Kozhemyakina, Lassar and Zelzer, 2015). *Col10a1* expression is first detected in the metacarpals of controls forelimbs around E13 (Figure 5C). At E13.5, its expression is stronger in metacarpal and is also strongly expressed in the center of the first phalanx, while at E14.5, it starts to be detected also in the second phalanx (Figure 5C). At these three stages, *Col10a1* expression is clearly downregulated in *Aldh1a2* mutant autopod, particularly in the latest forming structures (Figure 5C). These results show that chondrocyte differentiation is delayed in *Aldh1a2* mutant. This is consistent with the fact that the subsequent steps of endochondral ossification are delayed, and that newborn display smaller digits with visually smaller primary ossification center (Figure 5A). Together, these results suggest that *Aldh1a2* inactivation results in a delayed chondrocyte maturation, which eventually results in smaller ossification centers and smaller digits.

**FIGURE 6:**
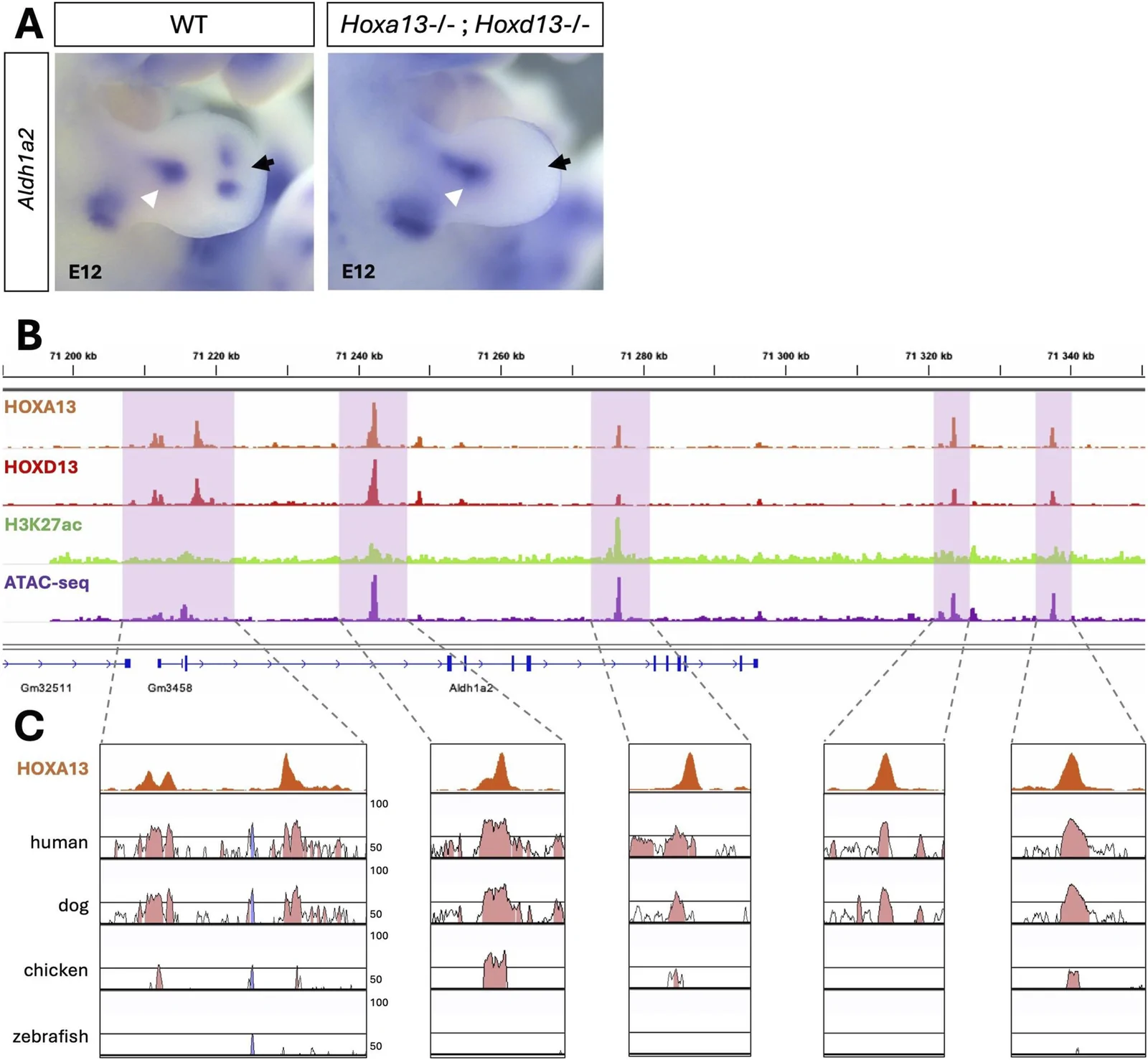
*Aldh1a2* expression relies on the function of *Hoxa13* and *Hoxd13*. **(A)** Whole mount *In Situ* Hybridization of *Aldh1a2* on WT and *Hoxa13/d13* double mutant forelimbs at E12 (n = 3). Black arrows highlight the distal limb in which *Aldh1a2* expression is lost, while the white arrowheads show the unaffected proximal expression. **(B)** IGV screenshot of HOXA13, HOXD13 and H3K27ac ChIP-seq (Sheth et al., 2016) and ATAC-seq signal from E11.5 WT distal forelimbs (Desanlis et al., 2020) at the *Aldh1a2* locus. **(C)** Conservation of the HOXA/D13-bound regions, highlighted in purple in **(B)**, with human, dog, chicken and zebrafish (from VISTA browser). Regions highlighted in red have more than 70% homology (over 100bp) with the mouse sequence.

### Aldh1a2 expression in the presumptive autopod likely relies on the function of Hoxa13 and Hoxd13

Compound heterozygous mutants for the *Hoxa13* and *Hoxd13* genes are characterized by soft tissue syndactyly of digit 2, three and 4 both in forelimbs and hindlimbs (Fromental-Ramain et al., 1996), suggesting that these genes play a role in the interdigital apoptosis process. Furthermore, it has been shown that both *Hoxa13* and *Hoxd13* individually modulate *Aldh1a2* expression in the distal limb (Kuss et al., 2009; Shou et al., 2013), and that HOXA13 probably directly controls its expression through several conserved binding sites at the *Aldh1a2* locus (Shou et al., 2013). To gain insights into the extent to which *Hox13* paralogs control *Aldh1a2* expression, we analyzed its expression in *Hoxa13;Hoxd13* double mutant embryos. Whole mount *in situ* hybridization shows that *Aldh1a2* expression is completely lost specifically in the presumptive autopod domain (Figure 6A, black arrow), where *Hox13* genes are normally expressed. Furthermore, ChIP-seq data of HOXA13 and HOXD13 from the distal limb cells show that these proteins are bound at several sites across the *Aldh1a2* locus, both close to the promoter, in introns and downstream of the gene (Figure 6B). Most of these sites are active regulatory elements, as shown by the presence of H3K27ac and chromatin accessibility (Figure 6B). These HOX13-bound sites are also conserved in mammals (as shown in human and dog) and to some extent in avian (shown in chicken) but are not conserved with more distant species like zebrafish (Figure 6C). While these results support a possible direct control of *Aldh1a2* expression by HOXA13 and HOXD13, the presumptive autopod of *Hoxa13* ^*−/−*^ *;Hoxd13* ^*−/−*^ embryos is smaller than the control and as a consequence, we cannot exclude that the loss of *Aldh1a2* distal expression that we show at E12.5 is associated with a delayed development of *Hoxa13* ^*−/−*^ *;Hoxd13* ^*−/−*^ distal limb bud and not a direct control of *Aldh1a2* expression by HOXA13 and HOXD13. However, we think this is unlikely as at E12.5 the presumptive autopod of *Hoxa13* ^*−/−*^ *;Hoxd13* ^*−/−*^ embryos resemble the presumptive autopod of E11.5 wild type embryos, a stage where *Aldh1a2* is clearly expressed in the distal limb bud (Figure 1A).

## Discussion

In this study, we investigate the mechanism by which RA signaling controls tissue regression in the interdigit domain by conditionally inactivating *Aldh1a2,* the gene encoding the enzyme that catalyzes the synthesis of RA in the presumptive autopod. We show that *Aldh1a2* conditional inactivation leads to a very severe soft-tissue syndactyly phenotype, which is caused by the loss of apoptosis in the interdigital tissue. Our transcriptomic analysis at E12.5 and E13.5 identified a small subset of downregulated genes associated with apoptosis in the developing limb. This includes the known RA targets *Bmp7* (Dupé et al., 1999) whose function in digit separation has been previously demonstrated (Kaltcheva et al., 2016) and *Mafb,* shown to trigger apoptosis in chick limbs when it is co-expressed with *cJun* (Suda et al., 2014). In addition, our study identified new RA targets that likely contribute to the severe syndactyly phenotype. The first one is *Adamts9*, a metalloproteinase of the extra-cellular matrix (ECM) whose inactivation leads to digit webbing (Dubail et al., 2014). Indeed, degradation of the ECM prevents proper cell attachment to the extra-cellular matrix, which is known to trigger anoikis, a specific form of apoptosis induced by integrin-dependent anchorage deficiency (Alanko et al., 2015). Interestingly, McCulloch and colleagues have shown that cleaved versican fragments generated by Adamts enzymes enable interdigital apoptosis when inserted in a mutant with no versican cleavage (McCulloch et al., 2009). This suggests that cells undergo apoptosis not because they lost the survival effect provided by the ECM but because they detected the presence of a peptide that is normally found in pro-apoptotic conditions. Sensing of the ECM degradation thus appears as an additional RA-dependent mechanism triggering apoptosis in the interdigital tissue. The second target is *Dkk1*, a Wnt inhibitor expressed in the interdigital tissue, whose inactivation results in impaired interdigital tissue regression (Mukhopadhyay et al., 2001). Consistent with this phenotype, *Dkk1* was shown to enhance the interdigital cell death mediated by BMPs in chick limbs (Grotewold and Rüther, 2002). -Of note, our results show that deregulation of *Dkk1* expression does not affect Wnt-mediated cell proliferation. The third target, *Adra2*, normally expressed in the interdigital tissue, was previously shown to accelerate apoptosis when induced in mesenchymal cell lines (Wang and Limbird, 1997). Finally, our RNA-seq data reveals *Fmo2* and *Fmo6* as downregulated genes upon *Aldh1a2* inactivation. Importantly, *Fmo2* expression is restricted to the interdigital tissue (GenePaint database) and is related to the generation of Reactive Oxygen Species (ROS, Siddens et al., 2014), which are another important player in apoptosis induction (Covarrubias et al., 2008; Salas-Vidal et al., 1998). Our results show that indeed *Aldh1a2* mutant limb bud are deprived of ROS.

While the downregulated genes mentioned above were previously shown to contribute to interdigital tissue regression by loss-of-function experiments, *Eya2* has only been reported to promote apoptosis in murine myeloid cells (Clark, Fee and Cleveland, 2002) and its function in the developing limb was unknown. We thus generated an *Eya2* mutant mouse line but the resulting homozygous mice have no sign of soft tissue syndactyly and exhibit a wild type limb phenotype. This shows that not all RAR-associated genes identified in our transcriptomic/genomic analysis are functionally required for interdigital apoptosis. Nonetheless, our results indicate that RA positively controls several pathways promoting apoptosis (Figure 7). Individually, each of these pathways, when inactivated, results in a relatively mild digital webbing as compared to *Aldh1a2* ^*c/c*^ autopod, thereby suggesting that these pathways likely have an additive or synergistic effect on interdigital tissue regression. This, however, does not preclude the existence of cross-regulation between some of these pathways. In support of this, it was shown that beads soaked in BMP grafted in the developing limb bud result in a rapid induction of *Dkk1* expression (Grotewold and Rüther, 2002).

**FIGURE 7:**
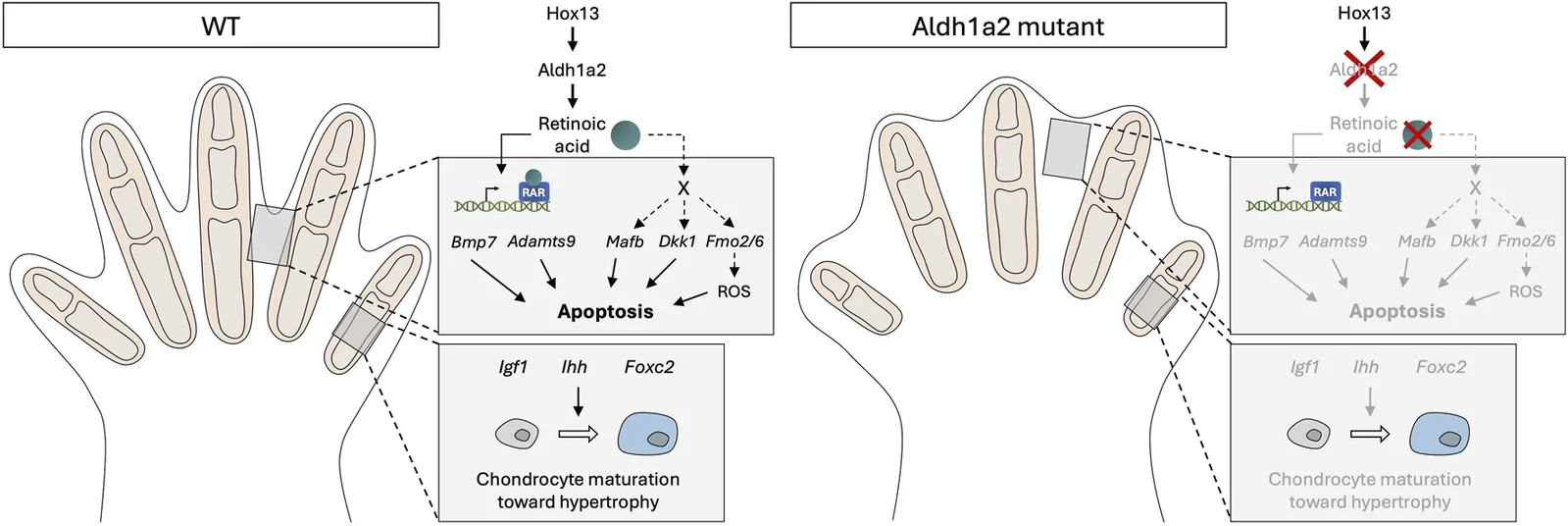
Model of the downstream effectors of RA signaling in the presumptive autopod domain. In the wild type distal limb (left panel), *Hoxa13* and *Hoxd13* genes regulate *Aldh1a2* expression. *Aldh1a2* synthesizes RA that will regulate the expression of several key genes, namely, *Bmp7*, *Adamts9*, *Mafb* and *Dkk1*, as well as the generation of ROS (likely through *Fmo2* and *Fmo6* function). This regulation could occur directly–through the RARs, and indirectly–through an unknown factor X. Altogether, these different pathways induce apoptosis, which results in interdigital tissue regression. In the digit area, RA signaling also indirectly regulates the expression of several genes important for the differentiation of hypertrophic chondrocytes. In *Aldh1a2* conditional mutant (right panel), RA is not synthesized and all the downstream effectors are downregulated. This leads to a complete absence of apoptosis in the interdigital tissue, resulting in soft tissue syndactyly, as well as impaired chondrocyte maturation, eventually leading to smaller digits.

Our approach to investigate the changes in activity of RAR targets upon *Aldh1a2* inactivation led to unexpected results. H3K27ac ChIP-seq analysis revealed only rare RAR targets showing significant (adjusted P-value <0.05) decrease in H3K27ac in *Aldh1a2* mutant limbs. As we used the entire distal limb bud to perform this ChIP-seq experiment and the interdigital tissue represents a rather small proportion of the total cell population, we thought that this cellular heterogeneity could explain that stringent conditions to assess significance may miss loci with reduced H3K27ac. We thus redid the analysis using a p-value <0.01. With the latter parameter, the decrease in H3K27ac expanded to RA targets associated with genes related to apoptosis. To ascertain which RA targets have actual reduced transcriptional activity in *Aldh1a2* mutant limbs, the characterization of RAR-bound loci at E13.5 and the isolation of interdigit tissue to perform H3K27ac ChIP-seq would be necessary.

One question that we could not address in this study is which apoptotic pathway, the intrinsic or the extrinsic (or both) is affected in *Aldh1a2* mutant. The Nomenclature Committee on Cell Death (NCCD) defines intrinsic apoptosis as a type of regulated cell death triggered by perturbations in either the extracellular or intracellular environment leading to the permeabilization of the outer membrane of mitochondria, release of cytochrome-c and activation of executioner caspases, mainly caspase-3 (Galluzzi et al., 2018). In contrast, extrinsic apoptosis is defined as being initiated by perturbations in the extracellular environment detected by receptors on the plasma membrane. These receptors include death receptors that get activated through the binding of their ligand, and the so-called dependence-receptors that get activated when their ligand drop below a certain threshold (Galluzzi et al., 2018). Our data reveal that RA does not regulate the expression of any component of the apoptosis cascade (except *Bid* and *Bax* which show only a minor deregulation, Supplementary Figures S3D, S3E), suggesting that the lack of apoptosis in *Aldh1a2* mutant result from a lack of activation of either the intrinsic and/or extrinsic apoptosis pathway rather than from the downregulation of its components. TNFs (Tumor Necrosis Factors) are inducers of the extrinsic pathway that bind death receptors at the cell surface to induce cell death (Galluzzi et al., 2018). During interdigital apoptosis, a specific TNF and its associated receptor (*FasL* and *Fas*) are found in the interdigital tissue (Svandova et al., 2017). In our transcriptomic analysis, *Tnfsf13b* was the only downregulated gene associated with TNF signaling. However, its expression was very low in control limb buds, and its known receptors, *Tnfrsf13b*, *Tnfrsf13c*, and *Tnfrsf17*, were not expressed in the limb (Desanlis, Paul and Kmita, 2020; RNA-seq data from this study). Thus, our transcriptomic data do not support a major contribution of *Tnfsf13b*-mediated signaling to RA-dependent interdigital apoptosis, although the involvement of other death receptor pathways cannot be excluded.

It is known that the classical apoptosis pathway mediated by caspases is responsible for interdigital cell death (Zuzarte-Luis et al., 2006). However, it has been shown that when using drug to inhibit caspase activity or in *Apaf-1* (a caspase-activating protein) mouse mutant, interdigital apoptosis still occurred (Chautan et al., 1999; Yoshida et al., 1998), suggesting that other caspase-independent mechanisms exist for the removal of the interdigital tissue. It was shown that lysosomal cathepsins act as a compensatory mechanism when caspases are inhibited, as a combined inhibition of both cathepsins and caspases led to an increase attenuation of cell death (Zuzarte-Luis et al., 2007). While we have not examined cathepsin activity in the *Aldh1a2* mutant, we found that ROS, which can induce the release of cathepsins in the cytosol where they trigger apoptosis (Voronina et al., 2024), is drastically reduced, suggesting that the defective interdigital tissue regression in *Aldh1a2* mutants may involve defective cathepsin function.

An additional role of RA in the distal limb was its effect on digit growth. This is consistent with a recent study from Duarte-Olivenza and colleagues showing that, in chick, pharmacological modulation of RA concentration impact upon digit size (Duarte-Olivenza et al., 2025). This effect could not be noticed in the previous studies in mice because of the embryonic lethality of the *Aldh1a2* and *Rdh10* mutant lines. In this study we show that the loss of RA signaling leads to a modest but progressive digit size reduction, which appears to be due to impaired chondrocyte maturation (Figures 5, 7). This raises the question of how RA might have an effect in the digit area as *Aldh1a2* expression is restricted to the interdigits and *Cyp26b1,* which degrades RA, is expressed in the digit condensations (reviewed in Chimal-Monroy et al., 2011). At E12.5 and E13.5, retinoic-acid receptor alpha (*Rara*) and gamma (*Rarg*) are expressed in the digit area, but in a domain wider than *Col2a1* (Supplementary Figure S6). It is thus possible that the RA signal could be integrated at the periphery of the digit condensation via *Rara/g*. This domain corresponds to the expression pattern of *Igf1* (van Kleffens et al., 1998), which is also downregulated at E12.5 and E13.5 in *Aldh1a2* mutant (Supplementary Figure S5A). Given that *Igf1* signaling can regulate *Ihh* (reviewed in Wang, Bikle and Chang, 2013), this provides a possible explanation as to how the lack of RA in the interdigital tissue affect the expression of key factors of digit development, such as *Ihh* and *Foxc2*.

The important role of RA signaling in the regression of the interdigital tissue raises the question of how *Aldh1a2* gets transcriptionally activated specifically in this domain. We show that the function of *Hoxa13* and *Hoxd13*, which are specifically expressed in the distal limb bud, is mandatory for *Aldh1a2* expression. However, whereas *Hoxa13* and *Hoxd13* genes are expressed across the entire autopod (Dollé et al., 1989), *Aldh1a2* is restricted to the interdigital space, raising the question of how this specific pattern is established. One possibility is that *Aldh1a2* expression is repressed in the digit area. Several lines of evidence support the hypothesis that *Sox9* mediates this repression. First, limb-specific conditional knock-out (with *Prx1*:Cre) of LSD1/KDM1a leads to elevated levels of *Aldh1a2*, suggesting that this enzyme represses *Aldh1a2* expression through its demethylase activity (Sun et al., 2020). Additionally, it has been shown that LSD1 binds close to *Aldh1a2* promoter (Sun et al., 2020) and that, in human chondrocytes, SOX9 directly interacts with LSD1 (Durand et al., 2020). Furthermore, ChIP-seq data from embryonic limbs also suggest that SOX9 binds close to *Aldh1a2* promoter (Garside et al., 2015; Yamashita et al., 2019). Finally, in a *Sox9* conditional mutant, apoptosis occurs in the entire autopod (Akiyama et al., 2002), which might be due to ectopic expression of *Aldh1a2* throughout the autopod. Thus, we propose that, in SOX9-expressing cells in the digit domain, SOX9 may recruit LSD1 to the *Aldh1a2* locus, thereby contributing to its repression and restricting *Aldh1a2* expression to the interdigital domain.

The requirement of RA signaling for interdigital apoptosis is known since almost 3 decades (Dupé et al., 1999; Rodriguez-Leon et al., 1999). However, *Aldh1a2* expression in the interdigital tissue is very poorly characterized across species with various autopod morphologies. To our knowledge, the only species (other than mouse) with separated digits and a reported *Aldh1a2* limb expression is the chicken. Similarly to the mouse, *Aldh1a2* is also detected in the interdigital tissue, albeit being less widely expressed (Díaz-Hernández et al., 2014). Another avian species with available data regarding interdigital apoptosis is the duck. In contrast to chicken, duck has webbed hindlimbs, which is associated with the anti-apoptotic effect of the Bmp antagonist *Gremlin* in the interdigital tissue (Merino et al., 1999). However, inactivation of BMPs in mice results only in partial interdigit tissue retention (Kaltcheva et al., 2016) suggesting that the webbed hindlimbs in duck is not solely due to the expression of *Gremlin. Aldh1a2* expression being unknown in duck limbs it would be interesting to investigate whether the lack of *Aldh1a2* expression also contributes to interdigit tissue retention in duck hindlimbs. Interestingly, digit webbing is not necessarily associated with lack of apoptosis as shown for the bat forelimb (Schindler et al., 2025). Mason and colleagues showed that *Aldh1a2* is expressed in the interdigital tissue of bat wings (Mason et al., 2015), in a pattern reminiscent of its expression in chick. It was initially reported that, similarly to the duck, the lack of interdigital apoptosis in the bat wing is mainly due to *Gremlin* expression in the interdigital tissue (Weatherbee et al., 2006). However, analysis of the developmental and cellular origins of the bat wing showed that interdigital tissue retention occurs despite the presence of interdigital apoptosis (Schindler et al., 2025). Indeed, the authors showed that apoptotic cells were detected in all wing interdigit and that the wing membrane develop from a specific fibroblast population that expressed a conserved gene program commonly used to pattern the proximal limb (Schindler et al., 2025). These studies from a very limited group of species suggest that interdigital tissue retention can arise from different mechanisms. Nonetheless, given the role of apoptosis in shaping the autopod morphology in different tetrapod species (Cooper et al., 2014), a more systematic study of the expression profile of RA-synthesizing enzymes would help better understanding the genetic mechanisms responsible for the diversity of autopod morphologies across the animal kingdom.

## Data Availability

The datasets presented in this study can be found in online repository with the following Gene Expression Omnibus accession number GSE335505 and GSE335520.
