# Supplementary material for: Molecular analysis of Aldh1a2 conditional inactivation in developing limbs reveals mechanisms of retinoic-acid mediated interdigital apoptosis
Source: Front Cell Dev Biol. 2026 Sep 21;14:1913173. doi: 10.3389/fcell.2026.1913173 (PMC13635163; doi:10.3389/fcell.2026.1913173)
Supplement: Supplementary file 1 [file DataSheet1.docx]

**Supplemental Figure legend**

**Figure S1**

**(A)** Hybridization Chain Reaction staining of *Aldh1a2* in E12.5 forelimbs of *Hoxa13^Cre/+^* controls and *Aldh1a2^c/c^* mutant.

**(B)** Exon count of *Aldh1a2* in our RNA-seq data of E12.5 distal forelimbs. Numbers highlighted in red indicate the floxed exon, which is fully deleted in *Aldh1a2^c/c^* mutants E12.5 forelimbs compared to *Hoxa13^Cre/+^* controls.

**(C)** P21 phenotype of forelimb in WT and *Aldh1a2^c/c^* mutant.

**(D)** P0 phenotype of forelimb in the different genotypes indicated.

**(E)** P0 phenotype of hindlimb in the different genotypes indicated.

**(F)** Cleaved caspase 3 staining in WT and *Aldh1a2^c/c^* mutant E13.5 forelimb.

**Figure S2**

**(A)** *In Situ* Hybridization showing the unaffected expression of *Bmp2* and *Fgf8* in the forelimb of E12.5 *Aldh1a2^c/c^* mutant.

**(B)** Top 10 terms from Gene Ontology analysis (ranked by smallest FDR) using the downregulated genes with a log2FC < -0.4. Genes belonging to the « bone development » category are indicated in bold.

**(C)** Hi-C data from E12.5 distal limbs (from Rodriguez-Carballo et al, 2017) shows enriched contact (black arrows) between the *Dhrs3* promoter and three RAR-bound site (highlighted in gray) located more than 100kb downstream.

**Figure S3**

**(A)** Top 10 terms with the smallest FDR from Gene Ontology analysis, using the downregulated genes with a log2FC < -0.4 (167 genes). Genes belonging to the « skeletal system development » category are indicated in bold.

**(B)** Venn diagram showing the direct targets of RA/RAR, identified by crossing the list of up- and downregulated genes from the RNA-seq at E13.5 (with |log2FC| > 0.4) to the list of genes identified from the ChIP peaks (presented in Figure 2D).

**(C)** Quantification of proliferative cells (Ki67-positive) in each interdigital domain (ID1 to 4) in *Hoxa13^Cre/+^* control and *Aldh1a2^c/c^* mutant E13.5 forelimbs. The ratio of Ki67+ cells represent the number of cells detected in the interdigital domain relative to its area. See material and methods for details. Statistical difference was determined by two-tailed Welch’s unequal t-test. ns: not significant.

**(D, E)** Heatmap showing the expression (RPKM normalized) of genes involved in interdigital tissue regression **(D)** and of genes from the intrinsic and extrinsic apoptosis pathway **(E)** in *Hoxa13^Cre/+^* control and *Aldh1a2^c/c^* mutant distal limbs at E13.5. Genes with a significantly differential expression (p-adj < 0.05) have their associated log2FC indicated.

**(F)** MA plot of the H3K27ac differential analysis between *Hoxa13^Cre/+^* control and *Aldh1a2^c/c^* mutant E13.5 distal limbs. Highly significant sites (p-adj < 0.05) are highlighted in red and sites significant only from the p-value (p < 0.01) are shown in blue. Significant sites associated to some of the relevant candidate genes are shown.

**Figure S4**

**(A)** *In Situ* Hybridization of *Eya2* in the forelimbs of E12.5 and E13.5 embryos in *Hoxa13^Cre/+^* control and *Aldh1a2^c/c^* mutant.

**(B)** IGV visualization of the RNA-seq at E12.5 and E13.5 in the distal limb at the *Eya2* locus showing the exons transcribed (top). The fourth exon targeted for the generation of the *Eya2* knockout is highlighted in purple. The bottom part shows the WT protein sequence of *Eya2* (the deleted exon is highlighted in purple), and the protein sequence in the *Eya2* mutant upon the deletion of exon 4 that creates premature stop codons (asterisks highlighted in red).

(C) Schematic of the exon 4 deletion in *Eya2*. Red lightnings indicate the CRISPR cutting sites. Primers used for genotyping are indicated. The genotyping PCR made from gDNA is shown for *Eya2^+/+^*, *Eya2^+/-^* and *Eya2^-/-^* individuals. The asterisk indicates an unspecific band.

(D) Western blot for EYA2 in WT and *Eya2-/-* limb protein extracts. Arrowhead indicates the protein loss at approximately 55kDa. Asterisks indicate unspecific bands.

**(E)** Forelimb phenotype from 2 weeks old pups of *Eya2*^+/+^ and *Eya2*^-/-^.

**(F)** Lysotracker staining on E13.5 forelimbs of *Eya2*^+/+^ and *Eya2*^-/-^ embryos. Scale bar is 250µm.

**Figure S5**

**(A)** Heatmap showing the expression (RPKM normalized) of genes belonging to the « bone development » and « skeletal system development » categories from the gene ontology at E12.5 (left) and E13.5 (right), respectively.

**(B)** Hybridization Chain Reaction staining of *Col2a1* in forelimbs of E12.5, E13.5 and E14.5 in *Hoxa13^Cre/+^* controls and *Aldh1a2^c/c^* mutant. Quantification of the digits size is shown on the right. P-values were determined by two-tailed Welch’s unequal variance t-test. n = number of limbs measured: control E12.5 n=8, mutant E12.5 n=8, control E13.5 n=6, mutant E13.5 n=6, control E14.5 n=6, mutant E14.5 n=6. ns: not significant; *: p-value < 0.01. Digit numbers are indicated in white. Scale bar is 200µm for E12.5 and E13.5 and 500µm for E14.5.

(**C**) Hybridization Chain Reaction staining of *Sox9* in *Hoxa13^Cre/+^* control and *Aldh1a2^c/c^* mutant E12.5 forelimbs. Scale bar is 200µm.

(**D**) Expression levels (RPKM normalized) of *Sox9* from the RNA-seq data from *Hoxa13^Cre/+^* control and *Aldh1a2^c/c^* mutant distal limb buds at E12.5 (left) and E13.5 (right). ns: not significant from the DESeq2 analysis.

**(E)** *In Situ* Hybridization of *Foxc2* in the forelimb of E13.5 embryos in *Hoxa13^Cre/+^* controls and *Aldh1a2^c/c^* mutant (n=2).

**Figure S6**

Hybridization Chain Reaction staining of *Col2a1* with either *Rara* or *Rarg* in E12.5 (top) and E13.5 (bottom) WT forelimb embryos. Scale bar is 200µm.
