# Supplementary material for: Molecular analysis of Aldh1a2 conditional inactivation in developing limbs reveals mechanisms of retinoic-acid mediated interdigital apoptosis
Source: Front Cell Dev Biol. 2026 Sep 21;14:1913173. doi: 10.3389/fcell.2026.1913173 (PMC13635163; doi:10.3389/fcell.2026.1913173)
Supplement: Supplementary file 2 [file Table2.docx]

**Supplementary Table 2. H3K27ac ChIP-seq versus RNA-seq. Table showing the regions with a significant adjusted p-value (<0.05) for the comparison of H3K27ac signal between *Hoxa13^Cre/+^* controls and *Aldh1a2^c/c^* mutant E13.5 distal forelimbs. The genes associated to the peaks are shown in the 3^rd^ column, and their expression fold change is indicated in the 4^th^ column when significant (NS = not significant). Coordinates highlighted in black correspond to regions bound by the RAR.**

| **Coordinate location H3K27ac ChIP-seq peak for Aldh1a2 control vs mutant (mm10)** | **Log2 fold change: H3K27ac ChIP-seq for Aldh1a2 control vs mutant** | **Associated gene(s) from GREAT (distance from the ChIP-seq peak is indicated)** | **Log2 fold change of the associated genes from RNA-seq Aldh1a2 control vs mutant E13.5** |
| --- | --- | --- | --- |
| chr9:82829224-82830675 | 0.820613244875689 | Irak1bp1 (+410) | 1.18 |
| chr16:28445059-28447315 | 0.781873357773795 | Fgf12 (-960) | NS |
| chr3:86541944-86549367 | 0.554258523867148 | Mab21l2 (+2627), Lrba (+320976) | 0.71, 0.26 |
| chr13:71958416-71963836 | 0.494190692973782 | Irx1 (+2597) | NS |
| chr8:107543346-107557380 | 0.477216971174495 | Psmd7 (+38101), Wwp2 (+113998) | NS, NS |
| chr15:50883220-50891656 | 0.431161348092978 | Trps1 (+1611) | 0.34 |
| chr1:19206000-19218997 | 0.37407934024265 | Tfap2b (+3585) | NS |
| chr10:125965139-125973365 | 0.337296270211662 | Lrig3 (+2999), Atp23 (+932096) | NS, NS |
| **chr3:30004145-30018787** | 0.322482638085414 | Mecom (+1690), Egfem1 (+928733) | NS, NS |
| **chr4:144998746-145001482** | -1.90772734383854 | Dhrs3 (+107037), Vps13d (+194891) | -1.35, NS |
| **chr14:16573888-16576063** | -1.75808494694378 | Rarb (+567) | -2.39 |
| **chr2:166030097-166032196** | -1.48032673541586 | Ncoa3 (+38510), Sulf2 (+124516) | -0.21, 0.22 |
| chr18:5186012-5187721 | -1.467845601325 | Zfp438 (+147572), Svil (+266327) | NS, NS |
| chr6:140688273-140690107 | -1.38015747595302 | Gm11077 (-40094), Aebp2 (+65501) | NS, -0.26 |
| **chr3:89086901-89088453** | -1.20021554852957 | Rusc1 (+5613), Ash1l (+137055) | -0.43, 0.31 |
| chr10:53434898-53435854 | -1.09260054847079 | Asf1a (-161576), Cep85l (-55429) | NS, NS |
| **chr6:140682192-140684679** | -1.06625147432063 | Gm11077 (-45848), Aebp2 (+59747) | NS, -0.26 |
| chr2:155871285-155874028 | -1.04074750981572 | Fam83c (-37803), Uqcc1 (+57653) | NS, NS |
| chr11:75047487-75048809 | -0.976544643623488 | Hic1 (+121371), Smg6 (+122325) | -0.55, NS |
| chr17:74866179-74868432 | -0.963175754928495 | Ltbp1 (-138262), Ttc27 (+149556) | NS, NS |
| chr6:140690533-140692531 | -0.92648379329557 | Gm11077 (-37752), Aebp2 (+67843) | NS, -0.26 |
| chr11:75052731-75055200 | -0.859004244269545 | Hic1 (+115553), Smg6 (+128143) | -0.55, NS |
| chr4:63052788-63057632 | -0.736157767099847 | Zfp618 (+89636), Ambp (+98963) | NS, NS |
| **chr4:98508252-98512964** | -0.704486112993302 | L1td1 (-216140), Patj (+114817) | NS, NS |
| chr11:75138731-75143360 | -0.689564371002338 | Hic1 (+28473), Smg6 (+215223) | -0.55, NS |
| chr9:75625138-75626577 | -0.59562466840994 | Lysmd2 (+117) | NS |
| chr11:75164877-75169592 | -0.571699511321728 | Hic1 (+2284), Smg6 (+241412) | -0.55, NS |
| chr4:144890879-144895662 | -0.516543158727876 | Dhrs3 (+194) | -1.35 |
